# Tdp-43 cryptic exons are highly variable between cell types

**DOI:** 10.1186/s13024-016-0144-x

**Published:** 2017-02-02

**Authors:** Yun Ha Jeong, Jonathan P. Ling, Sophie Z. Lin, Aneesh N. Donde, Kerstin E. Braunstein, Elisa Majounie, Bryan J. Traynor, Katherine D. LaClair, Thomas E. Lloyd, Philip C. Wong

**Affiliations:** 1 Departments of Pathology, Johns Hopkins University School of Medicine, Baltimore, MD 21205 USA; 2 Departments of Neuroscience, Johns Hopkins University School of Medicine, Baltimore, MD 21205 USA; 3 Departments of Neurology, Johns Hopkins University School of Medicine, Baltimore, MD 21205 USA; 4 Laboratory of Neurogenetics, NIA, NIH, Bethesda, MD 20892 USA; 5 Neural Development and Disease Department, Korea Brain Research Institute, Daegu, 701-300 South Korea; 6 Present address: Institute of Psychological Medicine and Clinical Neurosciences, Cardiff University School of Medicine, Cardiff, CF24 4HQ UK

**Keywords:** TDP-43 –Nonconserved cryptic exons, Bioinformatics, Amyotrophic lateral sclerosis, Frontotemporal dementia, Inclusion body myositis

## Abstract

**Background:**

TDP-43 proteinopathy is a prominent pathological feature that occurs in a number of human diseases including amyotrophic lateral sclerosis (ALS), frontotemporal dementia (FTD), and inclusion body myositis (IBM). Our recent finding that TDP-43 represses nonconserved cryptic exons led us to ask whether cell type-specific cryptic exons could exist to impact unique molecular pathways in brain or muscle.

**Methods:**

In the present work, we investigated TDP-43’s function in various mouse tissues to model disease pathogenesis. We generated mice to conditionally delete TDP-43 in excitatory neurons or skeletal myocytes and identified the cell type-specific cryptic exons associated with TDP-43 loss of function.

**Results:**

Comparative analysis of nonconserved cryptic exons in various mouse cell types revealed that only some cryptic exons were common amongst stem cells, neurons, and myocytes; the majority of these nonconserved cryptic exons were cell type-specific.

**Conclusions:**

Our results suggest that in human disease, TDP-43 loss of function may impair cell type-specific pathways.

**Electronic supplementary material:**

The online version of this article (doi:10.1186/s13024-016-0144-x) contains supplementary material, which is available to authorized users.

## Background

Recent genetic evidence has established the linkage between the neurological disorders amyotrophic lateral sclerosis (ALS) and frontotemporal dementia (FTD) [1–5]. The key pathological feature that is shared between ALS and FTD is the cytoplasmic aggregation and nuclear clearance of an RNA binding protein called transactive response DNA binding protein 43 kDa (TDP-43, *TARDBP*) [6]. Since the discovery of TDP-43, a number of other human diseases have also been characterized with TDP-43 pathology [7–12]. Of particular interest, however, is the pathogenesis of inclusion body myositis (IBM), which is believed to be primarily myogenic rather than neurogenic [13, 14]. To understand the mechanisms of disease pathogenesis that will inform appropriate therapeutic strategies, it will be critical to determine whether the pathways affected by TDP-43 proteinopathy differ between neurons and myocytes.

We have recently found that TDP-43 plays a major role in repressing nonconserved cryptic exons [15]. These cryptic exons are regions of the genome that are normally skipped by the spliceosome due to the presence of adjacent UG microsatellite repeats, the consensus binding site of TDP-43. When TDP-43 function is lost, these cryptic exons become activated and often lead to nonsense-mediated decay (NMD) of the associated mRNA. In our previous report [15], we utilized an *in vitro* inducible stem cell model of TDP-43 deletion. However, we have yet to establish the cell type-specific cryptic exons that arise *in vivo*. Here, we generated conditional Tdp-43 knockout mice to specifically delete Tdp-43 in excitatory neurons and skeletal myocytes. We found that Tdp-43 cryptic exons are highly variable between cell types and that many distinct pathways are altered—novel findings that have mechanistic and therapeutic implications for human diseases exhibiting TDP-43 proteinopathy.

## Methods

### Mouse breeding strategy

We crossbred our conditional *Tardbp* knockout mice (*Tardbp* ^F/+^) with *CamKIIa-Cre* transgenic mice to obtain a cohort of *CamKIIa-Cre*;*Tardbp* ^F/+^ mice which were subsequently crossbred to *Tardbp* ^F/+^ mice to generate the final cohort: *CamKIIa-Cre*;*Tardbp* ^+/+^, *CamKIIa-Cre*;*Tardbp* ^F/+^ and *CamKIIa-Cre*;*Tardbp* ^F/F^ mice. A similar strategy was applied when crossbreeding the *MLC-Cre* driver line to *Tardbp* ^F/+^ mice. All mouse experiments were approved by the Johns Hopkins University Animal Care and Use Committee.

### Histology and immunohistochemistry

For the *CamKIIa-Cre* line, wildtype and floxed mice were anaesthetized and perfused with 4% paraformaldehyde. Brains were embedded into paraffin, cut into 10 μm sections and stained according to standard protocols. For the *MLC-Cre* line, wildtype and floxed mice were anaesthetized and sacrificed by decapitation. Muscle tissue was then rapidly dissected and flash frozen in liquid nitrogen cooled isopentane. Frozen cryosections were cut at 10 μm thickness and stained according to standard protocols. Immunoreactivity was visualized using the Vectastain ABC Kit and diaminobenzidine peroxidase substrate (Vector Laboratories). Images were obtained using Olyumpus BX53 microscope.

### Immunoblot analysis

For the *CamKIIa-Cre* line, wildtype and floxed mice were anaesthetized and sacrificed by decapitation. Brain tissue was then rapidly dissected and manually homogenized in RIPA buffer (Sigma) containing an EDTA-free protease inhibitor cocktail (Thermo Scientific). For the *MLC-Cre* line, wildtype and floxed mice were also anaesthetized and sacrificed by decapitation. Muscle tissue was snap frozen in isopentane cooled with liquid nitrogen, manually ground into a powder, and then homogenized in RIPA buffer with protease inhibitor cocktail. Protein concentration was determined using the BCA assay (Pierce). Proteins were resolved using the NuPAGE 4-12% Bis-Tris Gel (Novex) with NuPAGE MES SDS Running Buffer (Novex), and transferred to PVDF membrane (Millipore) with NuPAGE Transfer Buffer (Invitrogen).

The following antibodies were used for protein blots, immunofluorescence, and immunohistochemical analyses: rabbit anti-TDP-43 (Proteintech 10782-2-AP and 12892-1-AP), anti-NeuN monoclonal antibody (Chemicon), anti-GAPDH monoclonal antibody (Sigma), Alexa Fluor 488-conjugated Donkey anti-Guinea Pig IgG (H + L) antibody (Jackson ImmunoResearch), Alexa Fluor 594- and 647-conjugated Donkey anti-goat and anti-rabbit IgG (H + L) antibodies (Life Tech.).

### RNA extraction, RNA-seq analysis

Total RNA was extracted from hippocampi of 3 month old female *CamKIIa-Cre*;*Tardbp* ^F/F^ (neuronal knockout) and littermate control mice (*CamKIIa-Cre*;*Tardbp* ^+/+^) using TRIzol (Life Tech.) and RNeasy Mini kits (Qiagen). Total RNA from 2 month old male *MLC-Cre*;*Tardbp* ^F/F^ (skeletal muscle knockout) and littermate control mice (*MLC-Cre*;*Tardbp* ^+/+^) was also extracted in a similar manner. For the *CamKIIa-Cre* line, 3 control brains and 3 knockout brains were analyzed and all mice were female. For the *MLC-Cre* line, 2 control quadriceps and 2 knockout quadriceps were analyzed and all mice were male. 100-bp paired end RNA-seq libraries were generated using Illumina Tru-seq kits and then sequenced on an Illumina HiSeq 2000. For RT-PCR analysis, total RNA was isolated using RNeasy Mini Kit (Qiagen). cDNA was synthetized using RevertAid First Strand cDNA Synthesis Kit (Thermo Scientific) with random primers. RNA-seq analysis was performed using HISAT [16] and Cufflinks [17] software suites and visualized on the UCSC Genome Browser [18]. Cryptic exons were identified as previously described [14]. To identify common pathways between species, gene ontology analysis was performed on cryptic exon targets using manual annotation of genes with known functions in combination with the bioinformatics resource DAVID v6.7 [19].

### RT-PCR primers

**Table:** 

Primer	Sequence	Tissue
Ap3b2-Forward	AGCCAGAATATGGCCACGAC	Neuron
Ap3b2-Reverse	CACTATGATGGGCACACGGA	Neuron
Camk1g-Forward	CTGGCCAAGATCACAGACTGG	Neuron
Camk1g-Reverse	CTGTGTAGACACCACGCTCT	Neuron
Sh3bgr-Forward	GGAGCAGAGGCTTGGATCAC	Muscle
Sh3bgr-Reverse	AAAGCCCACCACTTCTTGCT	Muscle
Tns1-Forward	CCTGGTCTATCAGCACTCCG	Muscle
Tns1-Reverse	GGGCTCCCGATTTCGTTCAT	Muscle

## Results

### Selective deletion of Tdp-43 in mouse excitatory neurons and skeletal myocytes

**Fig. 1:**
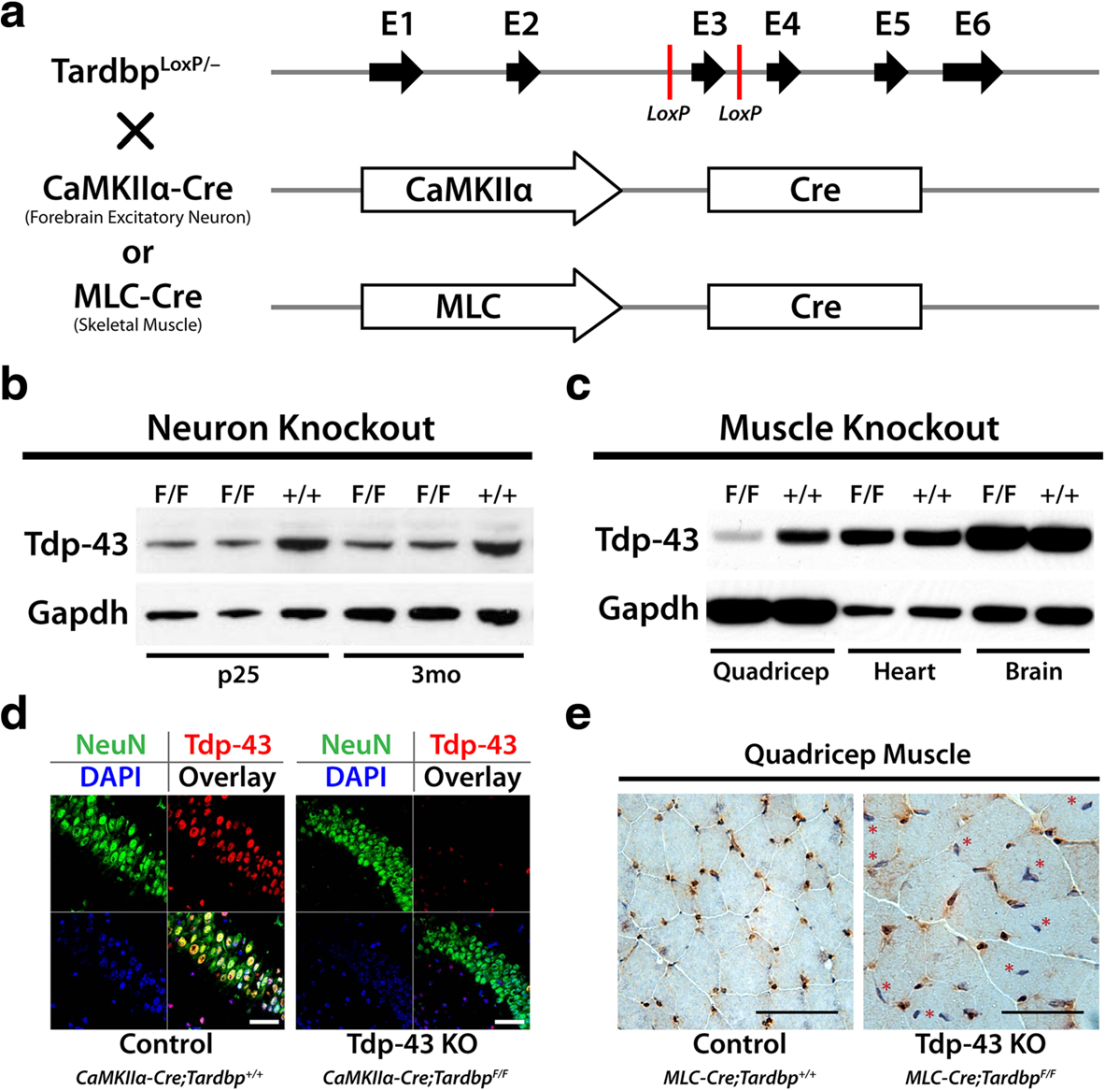
Generation of *CaMKIIα-Cre*;*Tardbp* ^*F/F*^ and *MLC-Cre*;*Tardbp* ^*F/F*^ knockout mice. (**a**) Breeding strategy to cross floxed *Tardbp* knockout mice with *CaMKIIα-Cre* or *MLC-Cre* mouse lines to conditionally delete Tdp-43 in excitatory neuron or skeletal muscle, respectively. Hippocampal protein extracts from *CaMKIIα-Cre*;*Tardbp* ^*F/F*^ knockout mice were taken from p25 and 3-month old mice, as indicated. Protein extracts from various muscle groups, as indicated, were taken from 2-month old *MLC-Cre*;*Tardbp* ^*F/F*^ mice. Immunoblotting confirms deletion of Tdp-43 in the hippocampi of *CaMKIIα-Cre*;*Tardbp* ^*F/F*^ knockout mice (**b**) and the quadriceps of *MLC-Cre*;*Tardbp* ^*F/F*^ knockout mice (**c**); biological replicates of immunoblotting were performed in excess of *n* = 3 to validate knockdown. (**d**) Immunofluorescence staining of hippocampal sections from 3 month old *CaMKIIα-Cre*;*Tardbp* ^*F/F*^ knockout mice demonstrate specific deletion of Tdp-43 from neurons (CA region, scale bar = 50 μm). (**e**) Immunohistochemical staining of Tdp-43 in quadriceps from 3 month old *MLC-Cre*;*Tardbp* ^*F/F*^ knockout mice also reveals loss of Tdp-43, as indicated by asterisks (scale bar = 50 μm)

To identify the cryptic exons repressed by Tdp-43 in neurons and myocytes, we utilized the Cre recombinase system to conditionally delete Tdp-43. Mice harboring floxed *Tardbp* knockout alleles [20] were crossbred with either *CaMKIIα-Cre* [21] or *MLC-Cre* [22] driver lines (Fig. 1a). The promoter of the calcium/calmodulin-dependent protein kinase II alpha subunit (*CaMKIIα*) drives expression primarily in the excitatory neurons of the cortex and hippocampus whereas the promoter of the myosin light chain 1/3 locus (*MLC*) drives expression in type II fast-twitch skeletal muscle fibers. Efficient deletion of Tdp-43 can be detected by immunoblot in brain (Fig. 1b) and skeletal muscle (Fig. 1c); residual Tdp-43 in F/F mice reflects the presence of other cell types that do not express *CaMKIIα-Cre* or *MLC-Cre*. Neuron specific deletion of Tdp-43 was confirmed by immunofluorescence staining of hippocampal sections (Fig. 1d); deletion of Tdp-43 in myocytes was also verified by immunohistochemistry (Fig. 1e).

### Identification of cryptic exons associated with Tdp-43 loss of function in neurons and myocytes

**Fig. 2:**
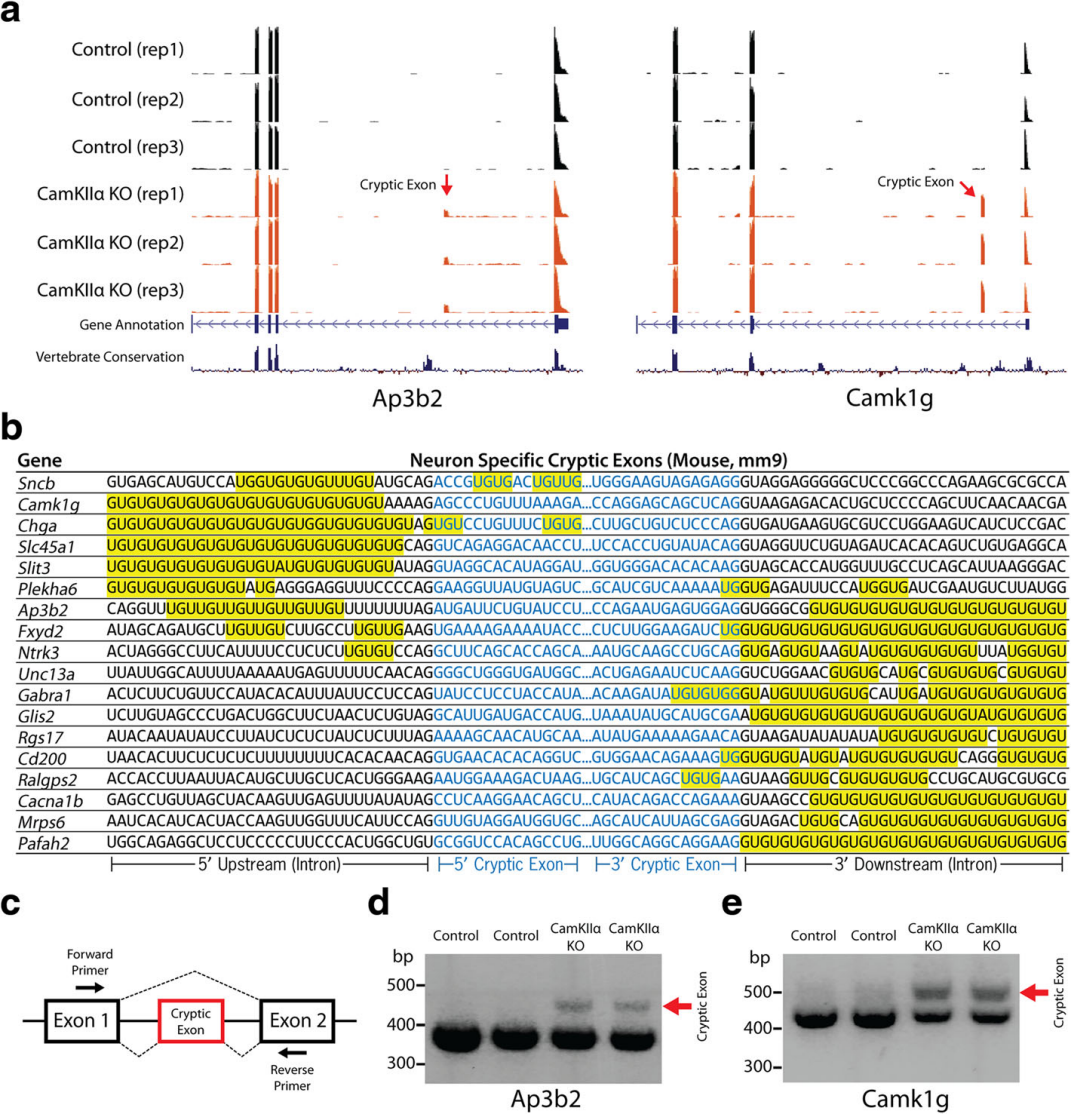
Neuron-specific cryptic exons (*CaMKIIα-Cre*;*Tardbp* ^*F/F*^ knockout mice). (**a**) Visual examples of neuron-specific cryptic exons (*Ap3b2*, *Camk1g*). (**b**) Neuron-specific cryptic exons are flanked by UG repeats that are present upstream, downstream or within the cryptic exon sequence itself. (**c** to **e**) RT-PCR validation of cryptic exons (red arrows) in RNA extracted from hippocampi of 3 month old *CaMKIIα-Cre*;*Tardbp* ^*F/F*^ mice. Refer to Additional file 2 for a complete list of cryptic exons

To identify the cryptic exons of mouse neurons, RNA-sequencing (RNA-seq) analysis was performed using RNA extracted from hippocampi of 3 month old *CaMKIIα-Cre*;*Tardbp* ^*F/F*^ mice and controls. Similar to our *in vitro* stem cell culture model of Tdp-43 deletion [15], we also found cryptic exons in the brains of *CaMKIIα-Cre*;*Tardbp* ^*F/F*^ knockout mice (Fig. 2a). Neuron-specific cryptic exons were still flanked by UG microsatellite repeats (Fig. 2b) and could be classified as standard cryptic exons, transcriptional start sites, exon extensions or premature polyadenylation sites (Additional file 1: Table S4, Additional file 1: Figure S1). Previously published CLIP data was also able to confirm the presence of a direct interaction with Tdp-43 (Additional file 1: Figure S2) [23]. Finally, to further validate our RNA-seq data, RT-PCR analysis was able to confirm the presence of cryptic exons in the genes *Camk1g* and *Ap3b2*. Longer PCR products, indicating cryptic exon inclusion, were detected in *CaMKIIα-Cre*;*Tardbp* ^*F/F*^ knockout but not control mice (Fig. 2c *-*e).

**Fig. 3:**
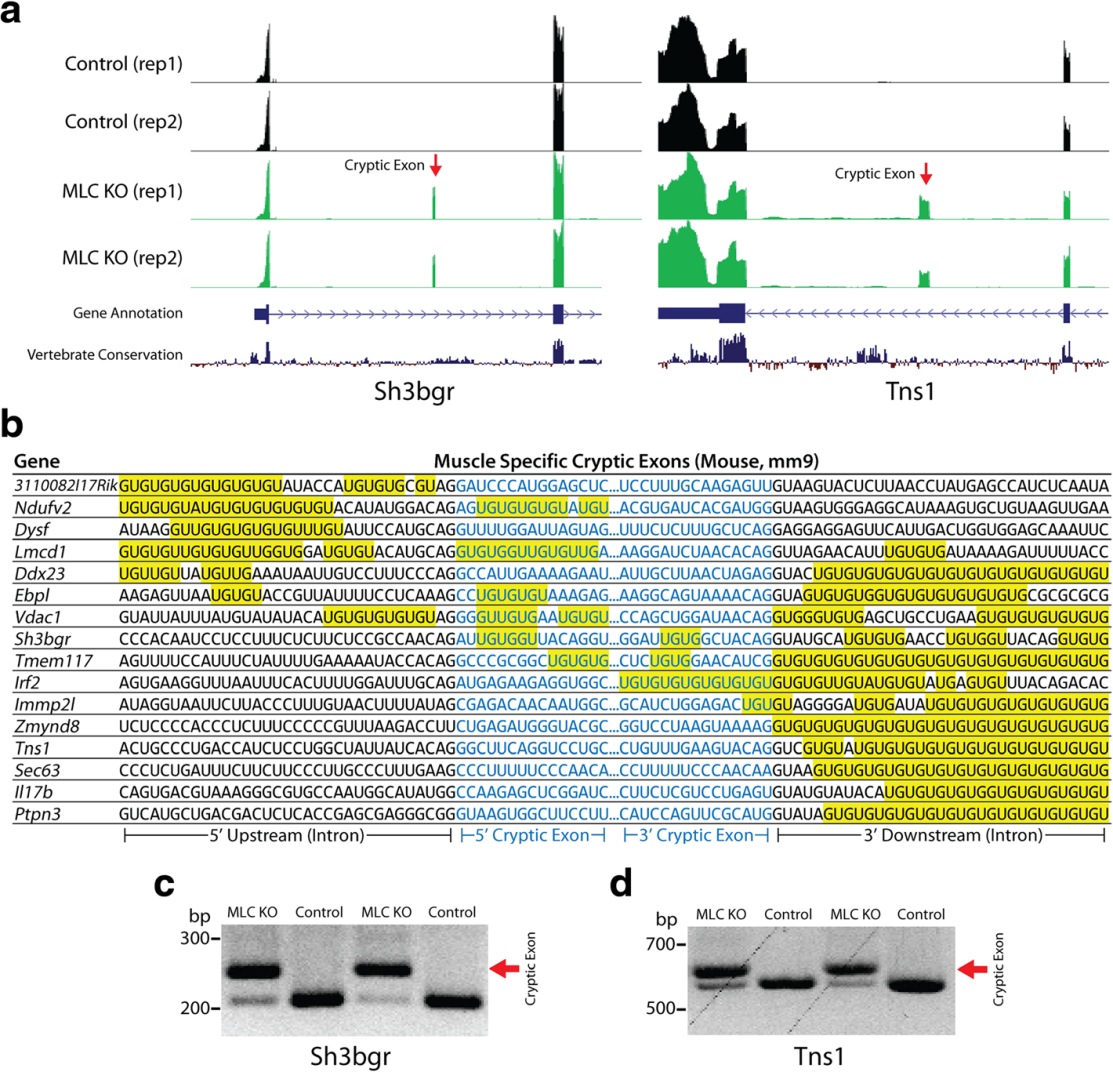
Muscle-specific cryptic exons (*MLC-Cre*;*Tardbp* ^*F/F*^ knockout mice). (**a**) Visual examples of muscle-specific cryptic exons (*Sh3bgr*, *Tns1*). (**b**) Muscle-specific cryptic exons are flanked by UG repeats that are present upstream, downstream or within the cryptic exon sequence itself. (**c** and **d**) RT-PCR validation of cryptic exons (red arrows) in RNA extracted from quadriceps of 2 month old *MLC-Cre*;*Tardbp* ^*F/F*^ mice. Refer to Additional file 2 for a complete list of cryptic exons

To determine whether cryptic exons of mouse myocytes would differ from those found in stem cells and neurons, we also performed RNA-seq analysis on quadriceps muscle from *MLC-Cre*;*Tardbp* ^*F/F*^ knockout mice and controls. Indeed, numerous muscle-specific cryptic exons could be identified (Fig. 3a). Furthermore, myocyte-specific cryptic exons were also flanked by UG microsatellite repeats (Fig. 3b); the presence of cryptic exons was confirmed by RT-PCR as shown for two genes, *Sh3bgr* and *Tns1* (Fig. 3c).

### Unique Tdp-43 cryptic exons occur in stem cells, neurons, and myocytes

**Fig. 4:**
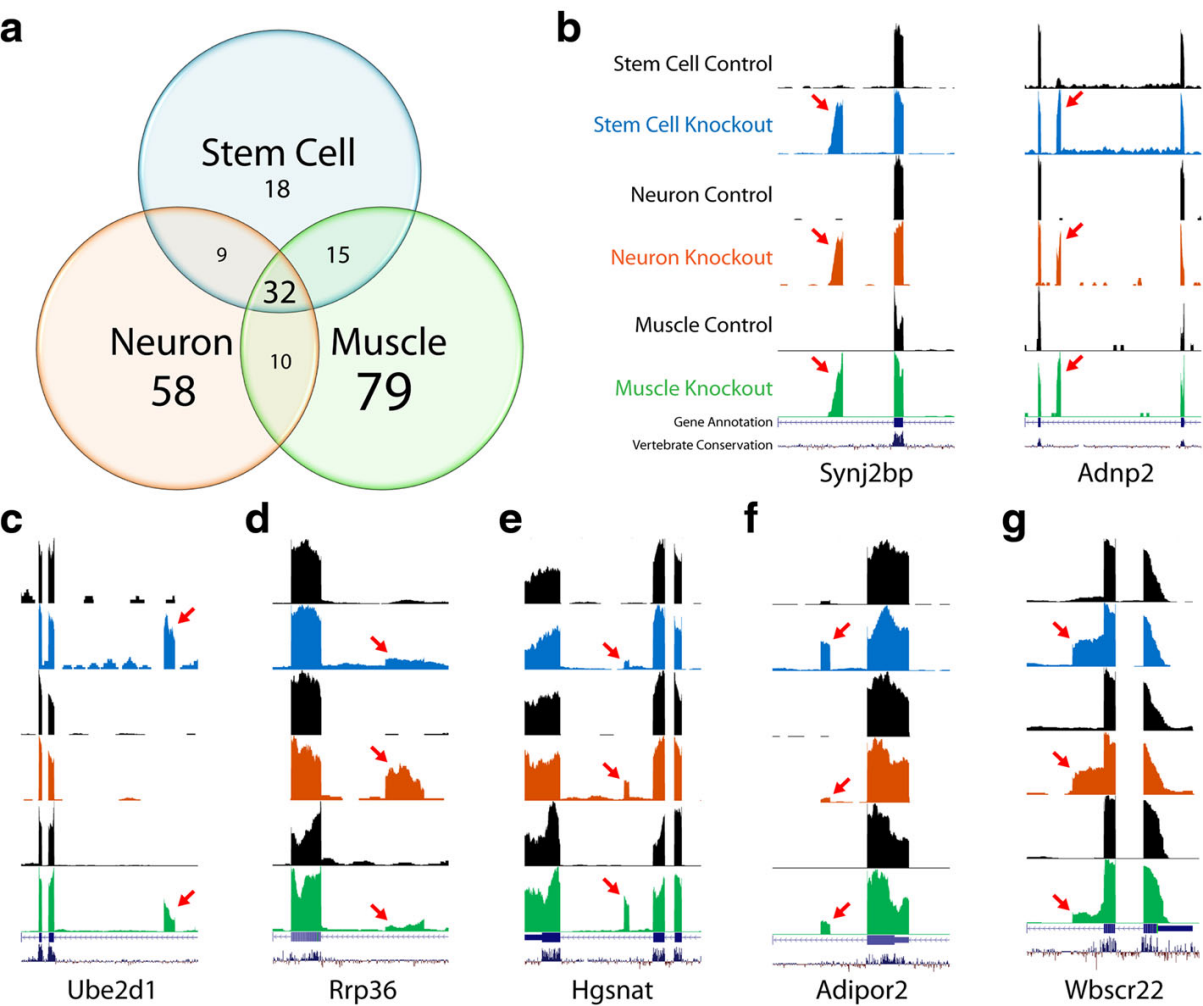
Tdp-43 cryptic exons are highly variable between cell types. (**a**) While some cryptic exons are common between cell types, many cryptic exons are unique to neurons (58), muscle (79) and stem cell [22]. Of the common cryptic exons, several are highly incorporated in mRNA regardless of splicing environment (**b**), while other cryptic exons are incorporated at varying levels depending on the cell type (**c** to **g**)

Having identified two new sets of cryptic exons belonging to mouse neurons and myocytes, we compared these sites with the cryptic exons previously identified in mouse stem cells [15]. Interestingly, only 66/221 (~30%) total cryptic exons showed any overlap between at least two cell types and only 32/221 (~14%) were common among all three cell types (Fig. 4a). Although the ratios varied, the majority of cryptic exons were unique to each individual cell type (155/221; ~70%). When normalized to the total number of cryptic exons in stem cells (74), neurons (109) and myocytes (136), the number of cell type-specific cryptic exons was lower in stem cells (18; ~24%) as compared to neurons (58; ~53%) and myocytes (79; ~58%). These results indicate that a large proportion of Tdp-43’s cryptic exons are cell type-specific (Additional file 1: Table S1 and S2).

Differential levels of cryptic exon incorporation, however, increase the complexity of these cryptic exon datasets. While certain cryptic exons, such as those in *Synj2bp* and *Adnp2*, can be observed at high levels in all three cell types (Fig. 4b), it is more common to see differential usage of cryptic exons amongst stem cells, neurons, and myocytes despite abundant transcription of the associated mRNA (Fig. 4c *-*g). For example, the cryptic exon in *Ube2d1* is highly incorporated in stem cells, moderately incorporated in myocytes, and absent in neurons (Fig. 4c). Conversely, the cryptic exon in *Rrp36* is high in neurons but low in stem cells and myocytes (Fig. 4d). Thus, it appears that the activation of a cryptic exon within a specific cell type depends not only upon transcription of the associated mRNA, but also the local splicing factor environment present within the cell (Additional file 1: Figure S3).

### Comparative analysis of genes affected by cryptic exon disruption

We have previously shown that Tdp-43’s nonconserved cryptic exons could disrupt gene function in cultured stem cells [15]. Similarly, while some neuron and myocyte cryptic exons reside in the 5’ or 3’ untranslated regions (~19%) with no clear effect on transcript levels, the majority of cryptic exons disrupt normal protein translation by introducing premature stop codons that lead to nonsense mediated decay (Additional file 1: Figure S4) or early termination of the mRNA transcript (~63%). Of these disrupted genes, numerous critical pathways are affected, ranging from mitochondrial function and protein regulation to transcriptional control and genome stability (Table 1). These findings demonstrate that cell type-specific pathways are altered when Tdp-43 function is lost and suggest that unique molecular pathways could differentially impact ALS-FTD and IBM.

**Table 1:**
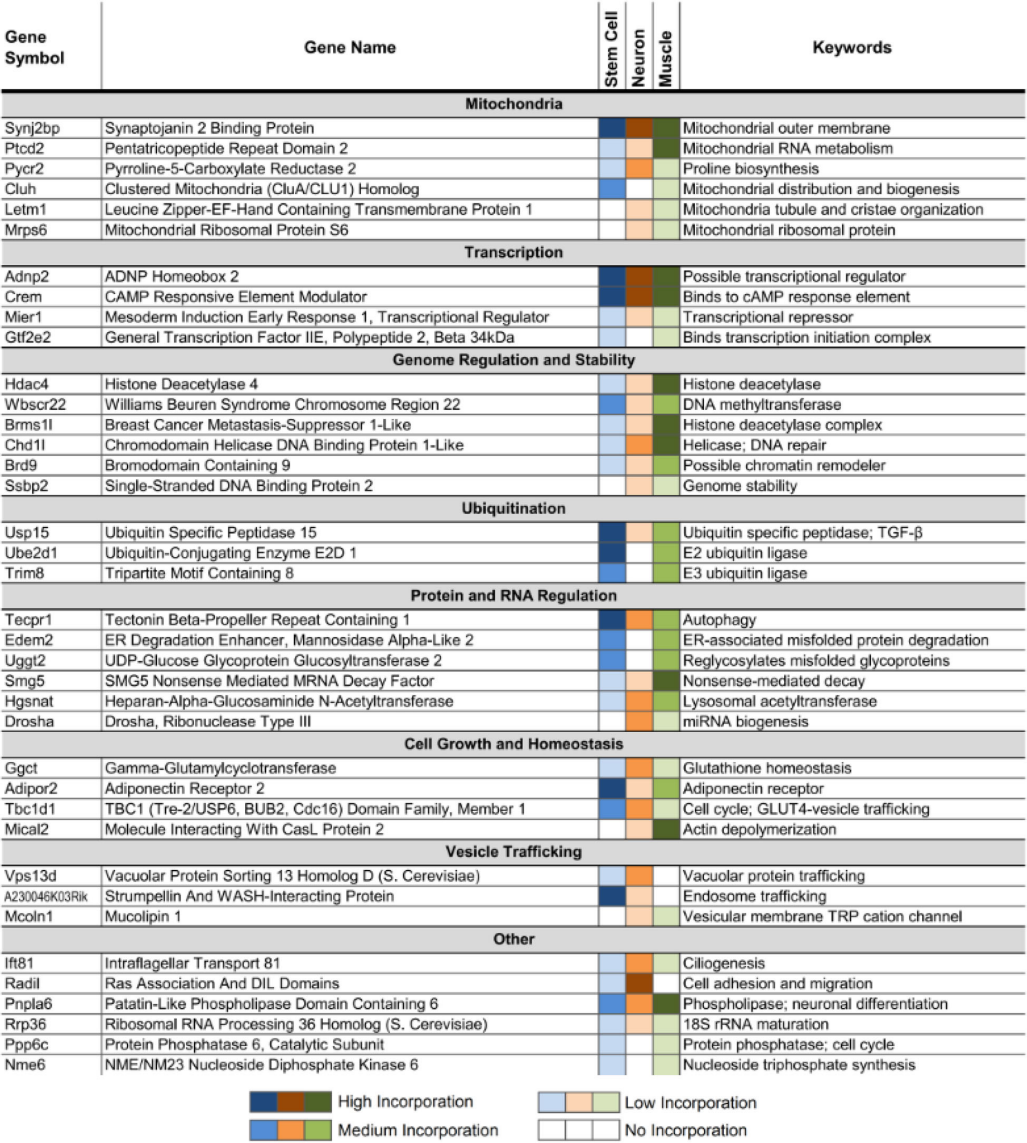
Common pathways affected by Tdp-43 cryptic exons across mouse stem cell, muscle and neuron (cryptic exon present in at least two cell-types)

Refer to Additional file 2 for a full list of cryptic exons

### Common pathways affected by Tdp-43 loss of function

Although many cryptic exons are predicted to induce nonsense mediated decay, their impact on mRNA and protein levels depends upon the frequency of cryptic exon incorporation. Across stem cells, neurons and myocytes, a broad group of genes are affected by Tdp-43 loss of function (Table 1). Many pathways are affected, from mitochondrial function and cell growth to transcription and genomic regulation, offering a possible explanation for the observed cell death associated with Tdp-43 deletion [24–28]; *CaMKIIα-Cre*;*Tardbp* ^*F/F*^ exhibit significant cortical atrophy at 8 months of age [12] while *MLC-Cre*;*Tardbp* ^*F/F*^ mice reach endstage by 4–5 months. Several other genes that are disrupted by cryptic exons also reflect previously reported observations: *Drosha* is involved in miRNA biogenesis [29], *Tecpr1* is involved in autophagy [30], and *Tbc1d1* and *Adipor2* are involved in fat metabolism [20].

Interestingly, a low percentage of cryptic exons (~6%) do not induce nonsense mediated decay, but still have an impact on protein structure. These cryptic exons do not contain any stop codons and have sequence lengths that are multiples of three, thereby preventing detrimental frameshifts (Additional file 1: Table S3). These inframe cryptic exons introduce short peptide insertions into the primary amino acid sequence of the protein, which may represent neoantigens.

## Discussion

We have found that Tdp-43’s nonconserved cryptic exons vary widely between cell types and affect many pathways that are critical for neuronal and muscle physiology. This suggests that in human disease, myogenic and neurogenic TDP-43 proteinopathies exhibit cell type-specific cryptic exons that could influence disease progression in unique ways. Although our RNA-seq data are based on a limited number of samples, future analysis to increase sample sizes would strengthen our findings. Identifying the cryptic exons that are specific to human neurons or myocytes will also help clarify the selective vulnerability associated with diseases such as IBM and ALS-FTD.

While it remains to be proven whether TDP-43 loss of function is a central driver of human disease, our data demonstrates that within neurons and myocytes, TDP-43 is the major splicing repressor for numerous nonconserved cryptic exons. In human disease, dysregulation of Tdp-43 function may impair other neuronal functions beyond mRNA splicing such as axonal trafficking, hyperexcitability, and liquid-liquid phase separation [31–34]. Nevertheless, mouse models of Tdp-43 have demonstrated that constitutive deletion of *Tardbp* results in embryonic lethality [24, 25, 35, 36]. Conditional depletion of *Tardbp* in adult mice also leads to metabolic deficits and premature death [20] and significant neurodegeneration [26, 37, 38]. Together, these studies demonstrate the importance of Tdp-43 for cell survival.

The current work clarifies the mechanisms of toxicity that underlie Tdp-43 loss of function in the context of cryptic exon repression [15], a finding that has been replicated by other groups [39–41]. Our results suggest that cryptic exons disrupt unique pathways depending on cellular context, although future studies are needed to understand the degree to which these splicing errors contribute to cell death. Furthermore, TDP-43 belongs to a family of proteins that repress cryptic exons, suggesting that these splicing factors perform a general function in the cell to maintain splicing fidelity [42]. Thus, loss of TDP-43 splicing repression contributes to cell death and the pathways affected by cryptic exon incorporation are likely to be relevant for disease pathogenesis.

The question then becomes, how do we prevent incorporation of nonconserved cryptic exons? Therapeutic strategies that aim to directly interfere with cryptic exon splicing (e.g. anti-sense oligonucleotides) will be difficult to envision due to the sizeable number of nonconserved cryptic exons per cell. Furthermore, because nonconserved cryptic exons are different between mouse and human, testing splicing modulators for human cryptic exons in animal models is essentially impossible. However, the general splicing repression function of TDP-43 is conserved. Thus, it may be possible to use mouse models of TDP-43 deletion to specifically test therapeutic strategies that rescue TDP-43 mechanism of action rather than directly targeting individual cryptic exons. One strategy would employ gene therapy to introduce designer splicing factors—chimeric proteins that would couple the UG binding domain of TDP-43 with non-aggregating splicing repressor domains [15]—into neurons or muscles. In principal, this approach would repress most of TDP-43’s nonconserved cryptic exons in a manner that would be species-independent.

If neuron loss or skeletal muscle degeneration can be attenuated, such a therapeutic strategy could be rapidly translated into the clinic. Moreover, the observation that cryptic exons can occasionally introduce inframe insertions into mRNA suggests that certain human TDP-43 cryptic exons could represent biomarkers for human disease. We envision the development of specific antibodies to detect neoantigens introduced by human inframe cryptic exons in CSF or blood from patients, serving as either diagnostic biomarkers or tools to monitor the efficacy of treatments in future clinical trials.

## Conclusions

This study demonstrates that Tdp-43 represses a unique set of cryptic exons, depending on cellular context. Thus, the pathways impacted by Tdp-43 loss-of-function and cryptic exon incorporation are likely distinct for each cell type. These results have important implications for human disease, given that Tdp-43 proteinopathy can manifest in various tissues.

## Additional files

Additional file 1: Supplemental figures and tables. (PDF 4449 kb)
Additional file 2: Cryptic Exon Data Table. (XLSX 59 kb)

## Abbreviations

ALS: Amyotrophic lateral sclerosis
CaMKIIα: Calcium/calmodulin-dependent protein kinase II alpha
FTD: Frontotemporal dementia
IBM: Inclusion body myositis
MLC: Myosin light chain 1/3 locus
NMD: Nonsense-mediated decay
TDP-43: Transactive response DNA binding protein 43 kDa.
