# Supplementary material for: Tdp-43 cryptic exons are highly variable between cell types
Source: Mol Neurodegener. 2017 Feb 2;12:13. doi: 10.1186/s13024-016-0144-x (PMC5289002; doi:10.1186/s13024-016-0144-x)
Supplement: Additional file 1: — Supplemental figures and tables. (PDF 4449 kb) [file 13024_2016_144_MOESM1_ESM.pdf]

Supplemental Data

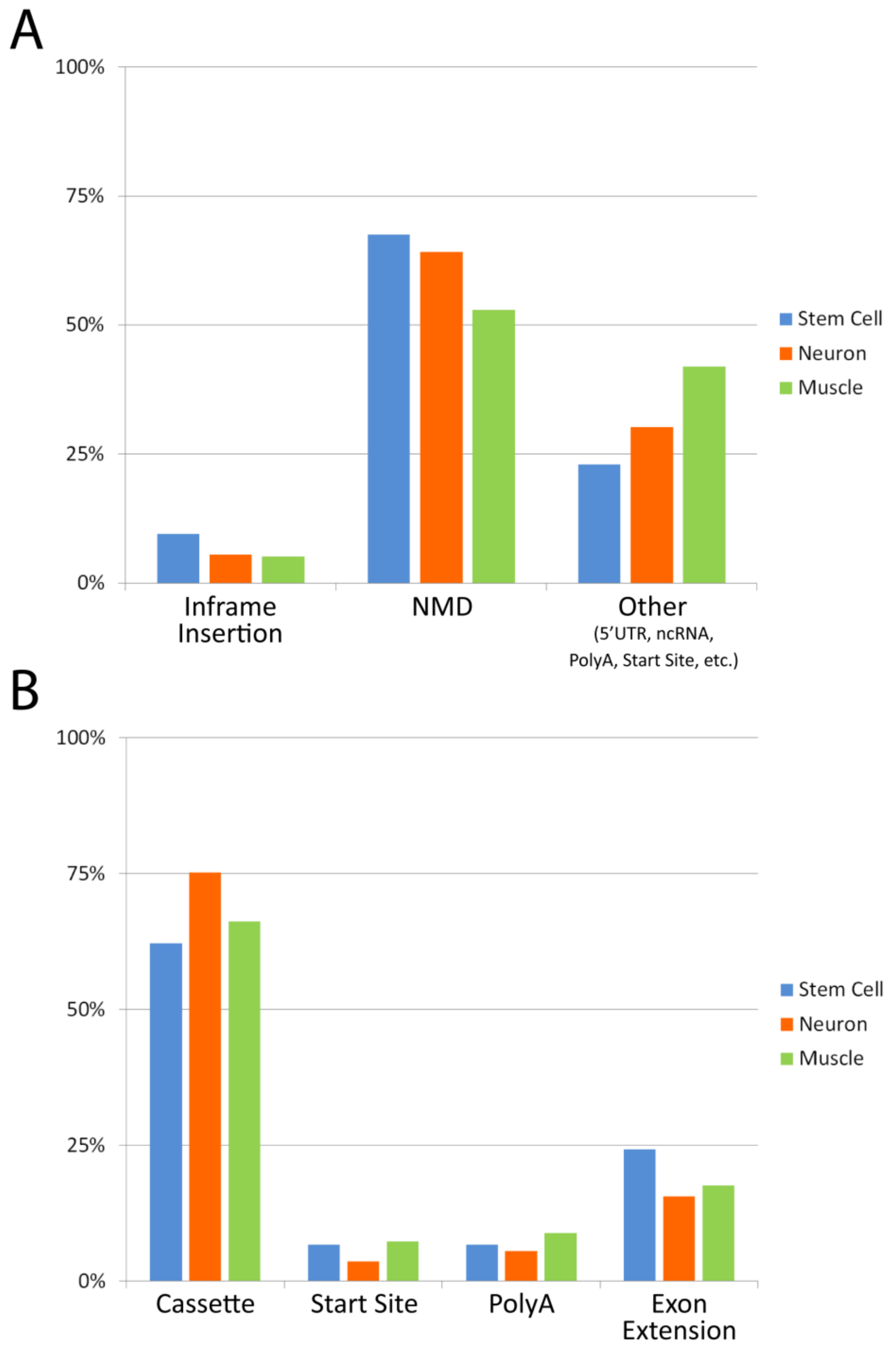

Fig. S1. Classification of stem cell, neuron and muscle cryptic exons associated with Tdp-43 loss of function. (***A***) Functional effect of cryptic exons on mRNA. (***B***) General grouping of cryptic exons (cassette exons, alternate start sites, alternate polyadenylation sites, and exon extensions—i.e. alternate 5' or 3' splice site utilization).

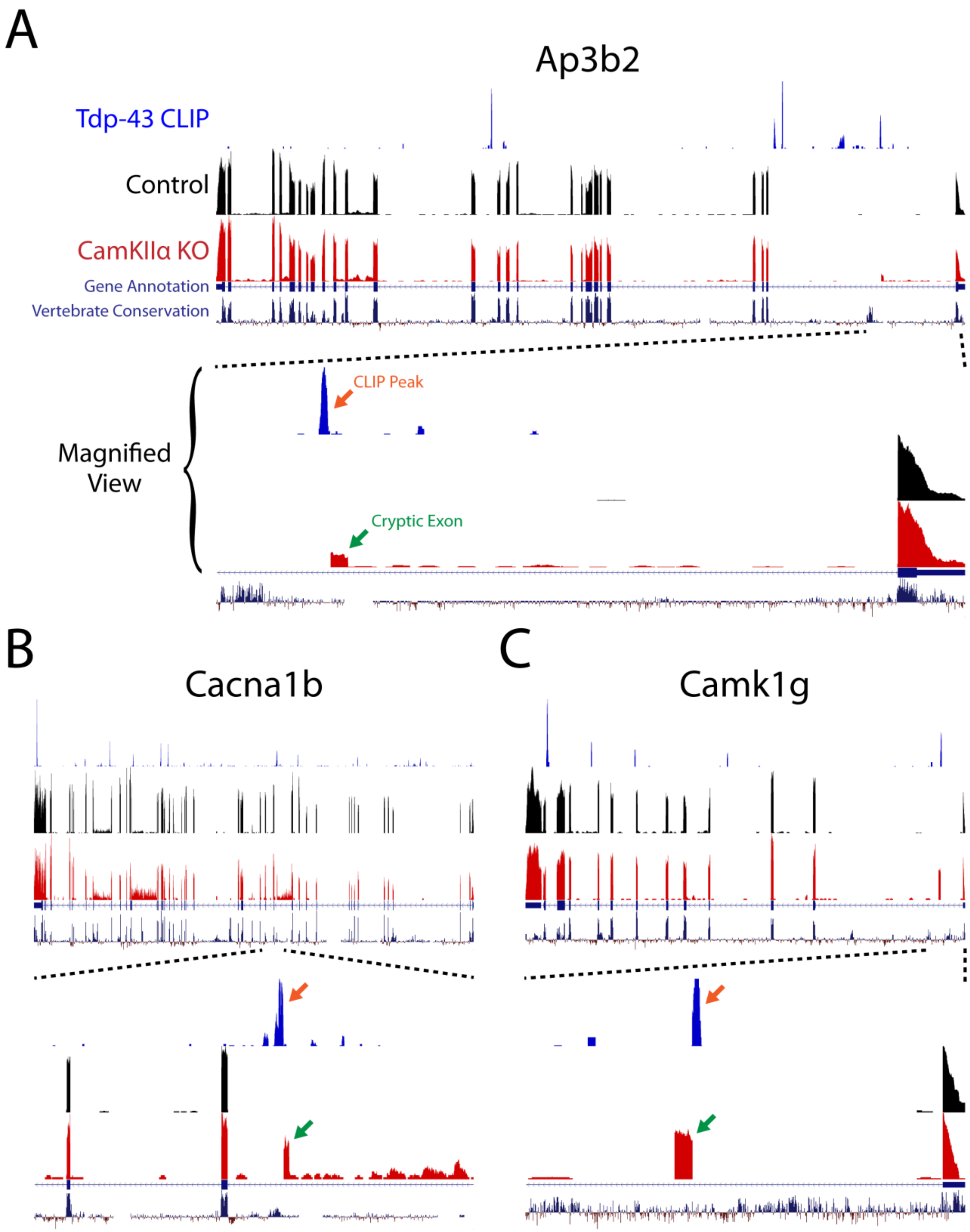
A
Ap3b2
Tdp-43 CLIP
Control
CamKIIα KO
Gene Annotation
Vertebrate Conservation
CLIP Peak
Magnified View
Cryptic Exon
B
Cacna1b
C
Camk1g

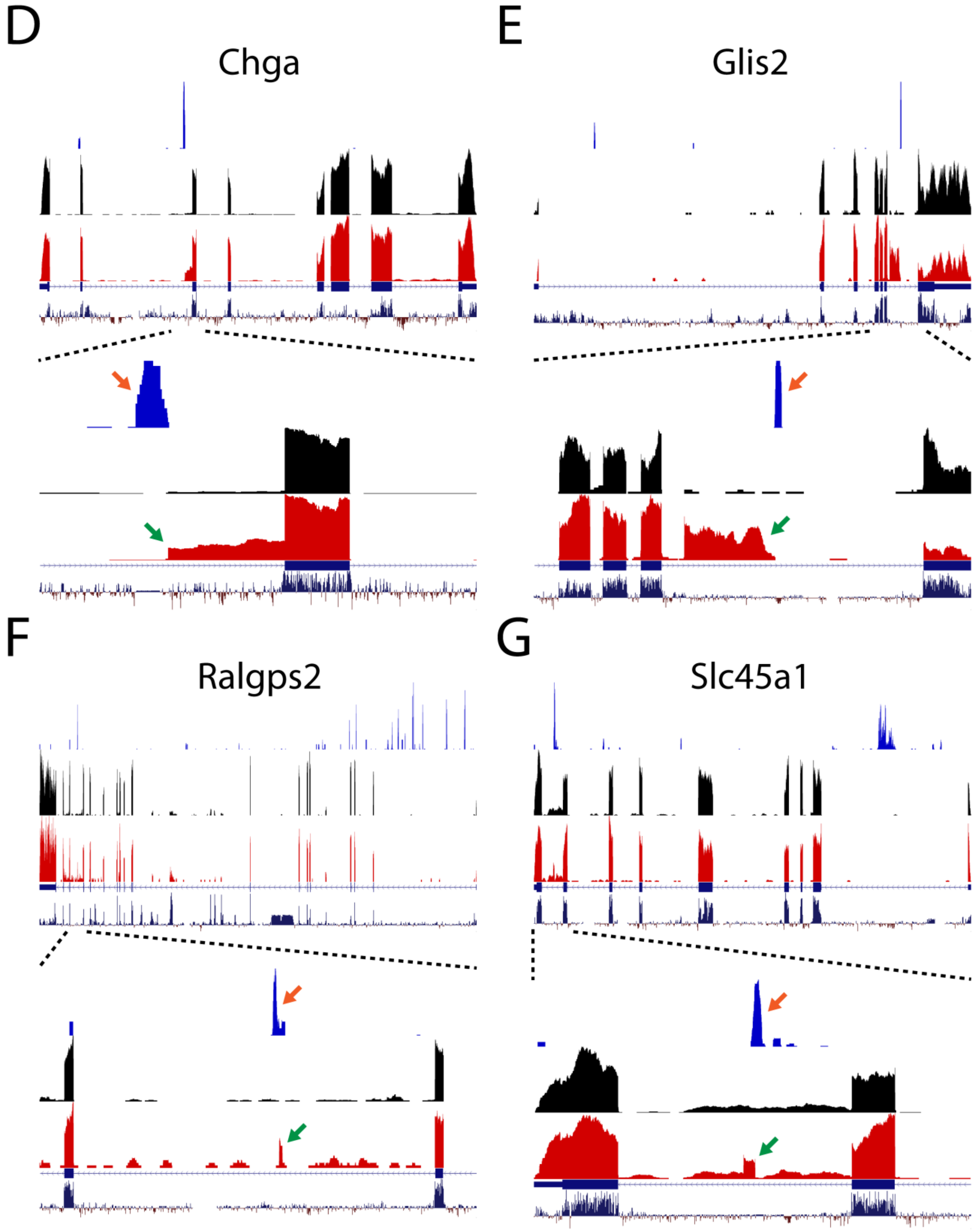

Fig. S2. HITS-CLIP peaks correlate with cryptic exons identified in *CaMKIIα-Cre*;*Tardbp*$^{F/F}$ knockout mice. HITS-CLIP was previously performed to identify Tdp-43's direct targets in mouse brain (18). (***A*** to ***G***) HITS-CLIP clusters (orange arrows) are adjacent to Tdp-43 cryptic exons (green arrows).

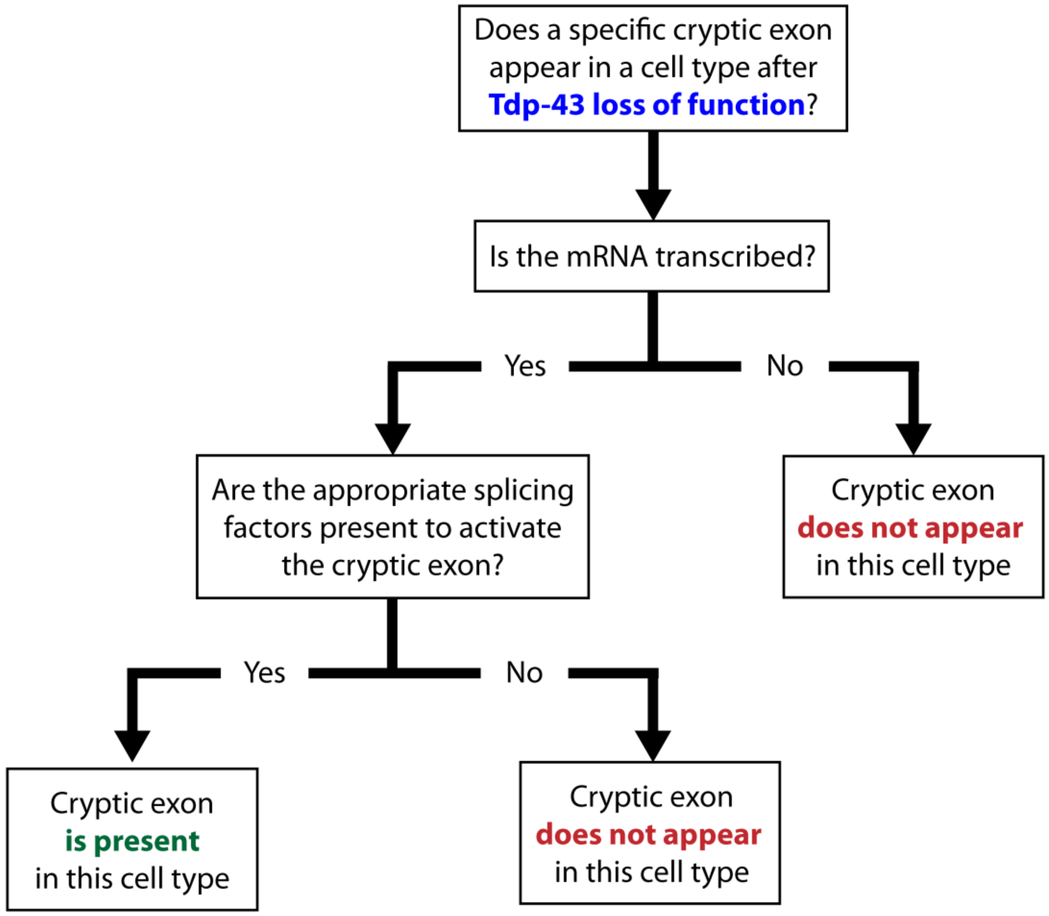

Fig. S3. Putative flow chart that governs whether a cryptic exon appears in a specific cell type.

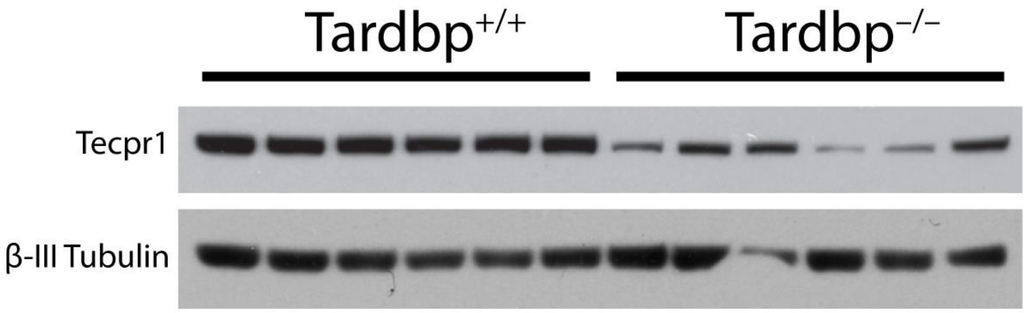

Fig. S4. Immunoblot with whole brain extracts from 8mo old female *CamKIIa-Cre*;*Tardbp*$^{F/F}$ knockout mice and controls. After Tdp-43 deletion, the incorporation of a cryptic exon in Tecpr1 leads to nonsense-mediated decay and a reduction in the protein levels of Tecpr1.

Gene Symbol	Gene Name	Stem Cell	Neuron	Muscle	Keywords
Pafah2	Platelet-Activating Factor Acetylhydrolase 2, 40kDa		High		Phospholipid lipase; metabolism
Cacna1b	Voltage-Gated Calcium Channel Subunit Alpha Cav2.2		High		Voltage-sensitive calcium channel subunit
Crlf1	Cytokine Receptor-Like Factor 1		High		Cytokine receptor subunit; neuronal survival and development
Fxyd2	FXYD Domain Containing Ion Transport Regulator 2		High		Sodium ATPase subunit gamma
Glis2	GLIS Family Zinc Finger 2		Medium		Transcription factor; neuron differentiation
Ralgps2	Ral GEF With PH Domain And SH3 Binding Motif 2		Medium		Guanine nucleotide exchange factor; cytoskeletal organization
Slit3	Slit Guidance Ligand 3		Medium		Axon guidance
Cluh	Clustered Mitochondria (CluA/CLU1) Homolog		Medium		Mitochondrial distribution and biogenesis
Tctn2	Tectonic Family Member 2		Medium		Hedgehog signaling
Slc45a1	Solute Carrier Family 45, Member 1		Medium		pH dependent glucose uptake
Rfx1	MHC Class II Regulatory Factor RFX		Medium		Regulator of MHC class II gene expression
Unc13a	Unc-13 Homolog A (C. Elegans)		Medium		Neurotransmitter release
Ank3	Ankyrin 3, Node Of Ranvier (Ankyrin G)		Medium		Regulates nodes of Ranvier and axonal initial segments
Uggt2	UDP-Glucose Glycoprotein Glucosyltransferase 2		Medium		Reglucosylates unfolded glycoproteins; protein quality control
Numbl	Numb Homolog (Drosophila)-Like		Low		Neurogenesis; neural progenitor cell maintenance
Pitpnm3	PITPNM Family Member 3		Low		Membrane lipid transfer
Kalrn	Kalirin, RhoGEF Kinase		Low		Rho guanine nucleotide exchange factor; synapse remodeling
Rasal1	RAS Protein Activator Like 1 (GAP1 Like)		Low		Suppressor of Ras signaling; cell proliferation and differentiation
Meis2	Meis Homeobox 2		Low		Homeobox transcription factor
Celf5	CUGBP, Elav-Like Family Member 5		Low		RNA-binding protein; pre-mRNA splicing
Kcnmb4	Big Potassium Channel Beta Subunit 4		Low		Calcium activated potassium channel regulatory subunit
Ntrk3	Neurotrophic Tyrosine Kinase, Receptor, Type 3		Low		Neurotrophin signaling
Ap3b2	Adaptor-Related Protein Complex 3, Beta 2 Subunit		Low		Neuron-specific AP3 vesicle-coat protein complex
Chga	Chromogranin A		Low		Neuroendocrine secretory protein
St6galnac5	GalNAc Alpha-2,6-Sialyltransferase V		Low		Cell surface sialyltransferase
Mrps27	Mitochondrial Ribosomal Protein S27		Low		Mitochondrial ribosomal protein
Abr	Active BCR-Related		Low		GTPase-activating protein; vestibular morphogenesis
Tub	Tubby Bipartite Transcription Factor		Low		Transcription factor; G protein signal transduction
Dlg3	Discs, Large Homolog 3 (Drosophila)		Low		NMDA receptor clustering; learning and synaptic plasticity
Aatk	Apoptosis-Associated Tyrosine Kinase		Low		Neuronal differentiation
Ppp3ca	Protein Phosphatase 3, Catalytic Subunit, Alpha Isozyme		Low		Calcium-dependent protein phosphatase
Cx3cl1	Chemokine (C-X3-C Motif) Ligand 1		Low		Chemotactic for leukocyte adhesion
Ak5	Adenylate Kinase 5		Low		Brain-specific adenylate kinase
Ssbp2	Single-Stranded DNA Binding Protein 2		Low		Genome stability
Ip6k1	Inositol Hexakisphosphate Kinase 1		Low		Inositol phosphokinase
Add2	Adducin 2 (Beta)		Low		Promotes assembly of spectrin-actin network

High Incorporation
Medium Incorporation
Low Incorporation
No Incorporation

Table S1. Neuron-specific cryptic exons impact a unique set of pathways (refer to Excel Data Table for list of cryptic exons).

Gene Symbol	Gene Name	Stem Cell	Neuron	Muscle	Keywords
Il17b	Interleukin 17B				T cell-derived cytokine, similar to IL17
Immp2l	Inner Mitochondrial Membrane Peptidase Subunit 2				Catalyzes removal of mitochondrial signal peptide sequence
Mad2l1bp	MAD2L1 Binding Protein				Regulator of late mitosis
Rhox3h	Reproductive homeobox 3H				Homeobox transcription factor
Dysf	Dysferlin				Congenital myopathies; Ca(2+) sensor; membrane regeneration and repair
Zbtb16	Zinc Finger And BTB Domain Containing 16				Transcription factor involved in cell cycle progression
Tns1	Tensin 1				Cell migration; focal adhesions and cytoskeleton mediated signal transduction
Sec63	SEC63 Homolog, Protein Translocation Regulator				Endoplasmic reticulum protein translocation
Irf2	Interferon Regulatory Factor 2				Interferon regulatory transcription factor
Chd1	ATP-Dependent Helicase CHD1				ATP-dependent chromatin remodeling; transcription
Plcl1	Phospholipase C-Like 1				Inositol phospholipid-based intracellular signaling; no phospholipase activity
Mlxipl	MLX Interacting Protein-Like				Transcriptional repressor
Scn4a	Sodium Channel Protein Type IV Subunit Alpha				Voltage-gated sodium channel
Tep1	Telomerase-Associated Protein 1				Telomerase ribonucleoprotein complex
Ptpn3	Protein Tyrosine Phosphatase, Non-Receptor Type 3				Tyrosine phosphatase; cytoskeletal regulation
Sgcd	Sarcoglycan, Delta				Dystrophin-glycoprotein complex; muscular dystrophy; dilated cardiomyopathy
Eif4g3	Eukaryotic Translation Initiation Factor 4 Gamma, 3				Probable translation initiation factor
Afap1l1	Actin Filament Associated Protein 1-Like 1				May be involved in podosome and invadosome formation
Lnpep	Leucyl/Cystinyl Aminopeptidase				Aminopeptidase that cleaves peptide hormones
Npepps	Aminopeptidase Puromycin Sensitive				Peptidase with broad specificity; regulates cell growth and viability
Sema3c	Semaphorin-E				Semaphorin receptor binding
Abcc9	Sulfonylurea Receptor 2				ATP-sensitive potassium channels; cardiomyopathy dilated type 1O
Pard3	Par-3 Family Cell Polarity Regulator				Asymmetrical cell division and cell polarization
Abhd16a	Abhydrolase Domain Containing 16A				MHC Class III; immunity
Nudt7	Nucleoside Diphosphate-Linked Moiety X Motif 7				Cleaves coenzyme A; Nudix hydrolase family
Coq10a	Coenzyme Q10A				Coenzyme Q chaperone; oxidative respiration
Epn1	Epsin 1				Clathrin vesicle binding; endocytosis
Rsu1	Ras Suppressor Protein 1				Ras signal transduction pathway
Pxn	Paxillin				Cytoskeletal protein; cell adhesion
Nxn	Nucleoredoxin				Redox-dependent negative regulator of Wnt signaling
Pgpep1	Pyroglutamyl-Peptidase I				Hydrolyzes N-terminal pyroglutamyl residues
Lmcd1	LIM And Cysteine-Rich Domains 1				Transcriptional cofactor
Trim55	Tripartite Motif Containing 55				Gene expression and protein turnover in muscle cells
Tnrc6b	Trinucleotide Repeat Containing 6B				RNA-mediated gene silencing
Cgrrf1	Cell Growth Regulatory Gene 19 Protein				Able to inhibit growth in several cell lines
Chac1	Cation Transport Regulator-Like Protein 1				Glutathione depletion; apoptosis initiation; Notch signaling
Erp44	Endoplasmic Reticulum Protein 44				Retention in early secretory pathway, endoplasmic reticulum
Trappc9	Trafficking Protein Particle Complex 9				Activator of NF-κB signaling
Nedd4	Cell Proliferation-Inducing Gene 53 Protein				E3 ubiquitin-protein ligase; lysosome degradation
Fto	Fat Mass And Obesity Associated				Dioxygenase; repair of alkylated DNA and RNA; body size and fat regulation
Ccndbp1	Cyclin D-Type Binding-Protein 1				Cyclin D; may negatively regulate cell cycle progression
Ndufv2	NADH-Ubiquinone Oxidoreductase 24 KDa Subunit				Core subunit of mitochondrial complex I
Acyp2	Acylphosphatase 2, Muscle Type				Acylphosphatase activity, membrane pumps (Ca2+/Mg2+-ATPase)

High Incorporation
Medium Incorporation
Low Incorporation
No Incorporation

Table S2. Muscle-specific cryptic exons impact a unique set of pathways (refer to Excel Data Table for list of cryptic exons).

Gene Symbol	Gene Name	Stem Cell	Neuron	Muscle	Keywords
Spata7	Spermatogenesis Associated 7				Retinal dystrophy; spermatogenesis
Spcs2	Signal Peptidase Complex Subunit 2 Homolog (S. Cerevisiae)				Microsomal signal peptidase complex
Hace1	HECT Domain And Ankyrin Repeat Containing E3 Ubiquitin Protein Ligase 1				E3 ubiquitin ligase; Golgi regulation
Kctd10	Potassium Channel Tetramerization Domain Containing 10				Cell proliferation
Fam96a	Family With Sequence Similarity 96, Member A				Chromosome segregation
Gsta4	Glutathione S-Transferase Alpha 4				Cellular detoxification
Tnfaip1	Tumor Necrosis Factor, Alpha-Induced Protein 1 (Endothelial)				Cytoskeleton structure; cell migration
Lrrc49	Leucine Rich Repeat Containing 49				Microtubule regulation
R3hdm2	R3H Domain Containing 2				Nucleic acid binding
Sh3bgr	SH3 Domain Binding Glutamate-Rich Protein				SH3 domain binding
Hps5	Hermansky-Pudlak Syndrome 5				Vesicular trafficking
Dnaja2	DnaJ (Hsp40) Homolog, Subfamily A, Member 2				Hsc70 co-chaperone
Psap	Prosaposin				Precursor for saposins A, B, C and D

High Incorporation
Medium Incorporation
Low Incorporation
No Incorporation

Table S3. Mouse cryptic exons predicted to introduce inframe insertions into mRNA.

Gene Symbol	UCSC ID	Cryptic Exon Location (mm9)	Cryptic Exon (bp)	Present in Stem Cell	Present in Neuron	Present in Myocyte	Inf. Ins.	UTR	Start Site	Poly A	Exon Ext.	Predicted NMD	Stem Cell Cryptic PSI	Neuron Cryptic PSI	Myocyte Cryptic PSI	Stem Cell CTRL (FPKM)	Stem Cell KO (FPKM)	Stem Cell CTRL vs KO (FC)	Neuron CTRL (FPKM)	Neuron KO (FPKM)	Neuron CTRL vs KO (FC)	Myocyte CTRL (FPKM)	Myocyte KO (FPKM)	Myocyte CTRL vs KO (FC)
Pnpla6	uc009krr.1	chr8:3524948-3525233	286	Y	Y	Y				Y		N	56	44	73	4.4	0.4	-11.3	3.7	3.0	-1.3	0.2	1.0	6.4
Tbc1d1	uc008xmm.1	chr5:64731056-64731165	110	Y	Y	Y						Y	64	44	22	0.5	0.1	-10.8	4.1	2.4	-1.7	3.2	0.7	-4.9
Usp15	uc007hgo.1	chr10:122588018-122588181	164	Y	Y	Y						Y	82	14	39	17.9	2.0	-9.1	5.4	3.7	-1.5	19.0	4.1	-4.7
Usp15	uc007hgo.1	chr10:122588018-122600648	12631	Y	Y	Y					Y	Y	86	14	39	17.9	2.0	-9.1	5.4	3.7	-1.5	19.0	4.1	-4.7
Adipor2	uc009dme.1	chr6:119319878-119319936	59	Y	Y	Y						Y	74	13	36	76.3	23.8	-3.2	14.5	15.2	1.0	18.9	11.0	-1.7
Chd1l	uc008qow.1	chr3:97395284-97395697	414	Y	Y	Y					Y	Y	4	52	75	23.0	9.8	-2.3	3.0	5.1	1.7	5.5	2.8	-2.0
Ptcd2	uc007rpm.1	chr13:100096403-100096525	123	Y	Y	Y						Y	23	11	77	34.7	14.9	-2.3	13.3	11.2	-1.2	16.7	5.4	-3.1
Slc7a6	uc009nfm.1	chr8:108692855-108693156	302	Y	Y	Y			Y			N	44	59	92	11.2	6.2	-1.8	2.9	2.8	-1.0	1.4	0.8	-1.7
Pycr2	uc007dwv.2	chr1:182834662-182834702	41	Y	Y	Y					Y	Y	13	49	5	124.4	77.0	-1.6	16.6	13.9	-1.2	9.8	6.2	-1.6
Tecpr1	uc009alj.1	chr5:144976607-144976659	53	Y	Y	Y						Y	98	35	52	2.9	1.9	-1.6	17.5	4.3	-4.1	3.4	0.9	-4.0
3110009E18Rik	uc007cjh.2	chr1:122022910-122022969	60	Y	Y	Y		Y				N	67	33	23	5.2	3.7	-1.4	1.4	1.5	1.1	4.2	0.9	-4.4
Rrp36	uc008ctu.1	chr17:46810105-46810288	184	Y	Y	Y						Y	13	26	9	35.1	25.1	-1.4	8.0	8.6	1.1	7.2	7.2	1.0
Wbscr22	uc008zxm.1	chr5:135539651-135539753	103	Y	Y	Y					Y	Y	35	23	40	64.3	46.6	-1.4	11.2	12.7	1.1	13.3	10.4	-1.3
Sars	uc008qzb.1	chr3:108245655-108245759	105	Y	Y	Y						Y	1	3	11	238.7	173.3	-1.4	48.0	43.3	-1.1	46.4	20.9	-2.2
Hgsnat	uc009lhg.1	chr8:27056421-27056468	48	Y	Y	Y						N	17	34	45	22.6	16.5	-1.4	16.7	18.8	1.1	9.5	6.6	-1.4
Ggct	uc009cah.1	chr6:54942069-54942165	97	Y	Y	Y						Y	5	47	32	24.3	18.1	-1.3	8.6	4.4	-1.9	7.2	1.7	-4.2
Brms1l	uc007npa.1	chr12:56964161-56966078	1918	Y	Y	Y					Y	Y	9	21	81	15.7	11.9	-1.3	23.6	11.3	-2.1	7.4	3.7	-2.0
Synj2bp	uc007oca.1	chr12:82610794-82611038	245	Y	Y	Y				Y		N	79	72	99	17.2	13.1	-1.3	15.5	13.6	-1.1	16.8	8.4	-2.0
2610507B11Rik	uc007kir.2	chr11:78077562-78077768	207	Y	Y	Y					Y	Y	26	1	30	55.0	42.8	-1.3	39.4	31.4	-1.3	69.5	33.4	-2.1
Pno1	uc007icc.1	chr11:17108384-17108667	284	Y	Y	Y						Y	9	2	11	89.0	72.6	-1.2	14.1	15.3	1.1	40.7	36.1	-1.1
Spata7	uc007ora.1	chr12:99872801-99872854	54	Y	Y	Y	Y					N	100	100	100	7.4	6.6	-1.1	6.3	4.6	-1.4	0.2	1.1	5.6
Brd9	uc007rej.1	chr13:74090298-74090335	38	Y	Y	Y						Y	23	17	36	11.5	10.5	-1.1	14.2	15.2	1.1	3.1	2.7	-1.1
Mier1	uc008txi.1	chr4:102834377-102834447	71	Y	Y	Y						N	11	19	30	10.2	9.5	-1.1	2.1	2.0	-1.1	3.3	3.1	-1.1
Smg5	uc008put.1	chr3:88144571-88144780	210	Y	Y	Y						Y	26	19	68	41.7	39.1	-1.1	16.2	16.3	1.0	11.0	22.9	2.1
Smg5	uc008put.1	chr3:88144571-88144685	115	Y	Y	Y						Y	33	24	68	41.7	39.1	-1.1	16.2	16.3	1.0	11.0	22.9	2.1
Hdac4	uc007cbf.1	chr1:94038097-94038158	62	Y	Y	Y						Y	26	20	100	1.0	1.0	-1.0	4.5	3.7	-1.2	2.1	4.6	2.2
Ift81	uc008zlm.2	chr5:123006915-123007000	86	Y	Y	Y						Y	29	41	28	5.7	5.5	-1.0	9.8	10.0	1.0	1.0	2.2	2.3
Adnp2	uc008fsk.1	chr18:80334892-80335043	152	Y	Y	Y						Y	96	76	100	27.7	28.2	1.0	5.6	7.3	1.3	0.9	2.8	3.1
Rtfdc1	uc008ocw.1	chr2:172290952-172291773	822	Y	Y	Y					Y	Y	2	5	1	33.0	33.7	1.0	28.0	26.3	-1.1	15.2	10.7	-1.4
Fam135a	uc007amk.2	chr1:24029346-24031051	1706	Y	Y	Y					Y	Y	55	29	49	1.3	1.4	1.1	1.1	2.0	1.8	2.9	1.7	-1.7
Spcs2	uc009imd.1	chr7:107004923-107005024	102	Y	Y	Y	Y					N	15	20	9	30.8	36.8	1.2	21.3	25.2	1.2	16.0	16.4	1.0
Crem	uc008dxl.2	chr18:3278079-3278238	160	Y	Y	Y			Y			N	100	68	80	0.0	0.4	29.5	2.4	0.9	-2.5	1.1	1.7	1.6
A230046K03Rik	uc007gkj.1	chr10:83023672-83023869	198	Y	Y							Y	80	20	--	29.1	9.1	-3.2	9.2	5.1	-1.8	--	--	--
Hace1	uc007fad.2	chr10:45359574-45359609	36	Y	Y		Y					N	51	30	--	10.5	7.7	-1.4	10.1	7.9	-1.3	--	--	--
Abr	uc007kft.1	chr11:76278083-76282079	3997	Y	Y							Y	32	4	--	8.7	6.5	-1.3	59.7	20.4	-2.9	--	--	--
Ift81	uc008zlm.2	chr5:123006942-123007000	59	Y	Y							Y	22	21	--	5.7	5.5	-1.0	9.8	10.0	1.0	--	--	--
Vps13d	uc012dox.1	chr4:144689255-144689366	112	Y	Y							Y	18	46	--	6.8	7.0	1.0	4.6	1.9	-2.4	--	--	--
Radil	uc009ait.1	chr5:142983773-142983862	90	Y	Y							Y	25	100	--	4.6	4.8	1.0	1.5	0.6	-2.5	--	--	--
Rap1b	uc007hdr.1	chr10:117253243-117253402	160	Y	Y			3'UTR				N	2	7	--	52.8	59.6	1.1	32.6	27.1	-1.2	--	--	--
Kctd10	uc008yzm.2	chr5:114826780-114826875	96	Y	Y		Y					N	34	33	--	11.1	18.5	1.7	15.7	16.3	1.0	--	--	--
Uggt2	uc007uzj.2	chr14:119399951-119400992	1042	Y	Y						Y	Y	68	30	--	6.0	13.5	2.3	2.1	4.2	1.9	--	--	--
Nme6	uc009rst.1	chr9:109740387-109740534	148	Y		Y						Y	19	--	27	25.7	12.0	-2.1	--	--	--	20.8	9.5	-2.2
Mettl10	uc009kcm.1	chr7:140026312-140026364	53	Y		Y						Y	8	--	63	21.9	12.5	-1.7	--	--	--	4.2	3.8	-1.1
Gtf2e2	uc009lki.2	chr8:34848447-34849451	1005	Y		Y				Y		N	32	--	8	45.9	29.5	-1.6	--	--	--	5.7	5.6	-1.0
Mettl6	uc007sxq.1	chr14:32302355-32302505	151	Y		Y						Y	10	--	15	20.7	15.9	-1.3	--	--	--	3.1	3.1	1.0
Tnks2	uc008hhu.2	chr19:36951510-36953724	2215	Y		Y					Y	Y	3	--	10	148.4	118.3	-1.3	--	--	--	32.9	24.5	-1.3
Ube2d1	uc007foo.2	chr10:70726333-70726465	133	Y		Y						Y	71	--	46	5.9	4.8	-1.2	--	--	--	193.8	116.2	-1.7
Ppp6c	uc008joi.1	chr2:39056407-39060352	3946	Y		Y					Y	Y	33	--	27	49.4	41.8	-1.2	--	--	--	27.4	23.5	-1.2
Cluh	uc007kca.1	chr11:74463197-74463680	484	Y		Y					Y	Y	37	--	20	13.7	11.6	-1.2	--	--	--	13.7	7.0	-2.0
Ppap2a	uc007rwr.1	chr13:113596569-113596634	66	Y		Y						Y	9	--	9	42.3	45.2	1.1	--	--	--	16.3	19.0	1.2
Fam96a	uc009qel.1	chr9:65985845-65985946	102	Y		Y	Y					N	2	--	8	55.3	60.7	1.1	--	--	--	44.1	38.4	-1.1
Lmbr1	uc008wug.1	chr5:29672685-29672822	138	Y		Y						Y	11	--	66	3.4	4.0	1.2	--	--	--	4.5	3.6	-1.2
Edem2	uc008nlg.2	chr2:155544126-155545223	1098	Y		Y					Y	Y	48	--	57	9.9	13.0	1.3	--	--	--	1.9	2.6	1.4
Pir	uc009uvl.2	chrX:160707610-160707645	36	Y		Y		Y			Y	N	45	--	38	14.1	20.4	1.4	--	--	--	3.9	2.5	-1.6
Trim8	uc008hts.1	chr19:46577504-46581418	3915	Y		Y					Y	N	57	--	37	28.4	45.3	1.6	--	--	--	7.3	7.9	1.1
Uggt2	uc007uzj.2	chr14:119399951-119400486	536	Y		Y						Y	34	--	35	6.0	13.5	2.3	--	--	--	0.1	0.6	5.8
Spata13	uc011zna.1	chr14:61321802-61321959	158	Y					Y			N	45	--	--	3.6	0.5	-6.9	--	--	--	--	--	--
Fam73a	uc008rte.2	chr3:151955521-151955675	155	Y								Y	52	--	--	13.4	4.1	-3.2	--	--	--	--	--	--
Atraid	uc008www.2	chr5:31354907-31355004	98	Y							Y	Y	18	--	--	40.1	21.4	-1.9	--	--	--	--	--	--
Mib1	uc008ebc.1	chr18:10769157-10769215	59	Y								Y	33	--	--	10.3	5.5	-1.9	--	--	--	--	--	--
Gsta4	uc009qty.2	chr9:78055004-78055045	42	Y			Y					N	9	--	--	596.2	326.6	-1.8	--	--	--	--	--	--
Lnp	uc008kdq.2	chr2:74372672-74372725	54	Y								Y	19	--	--	3.8	3.0	-1.3	--	--	--	--	--	--
Necap1	uc009dpt.1	chr6:122825542-122825633	92	Y								Y	11	--	--	35.3	31.2	-1.1	--	--	--	--	--	--
Snapc3	uc008tku.1	chr4:83083491-83083627	137	Y								Y	8	--	--	8.2	7.5	-1.1	--	--	--	--	--	--
Sulf1	uc007aia.2	chr1:12830579-12831017	439	Y							Y	Y	25	--	--	9.8	9.0	-1.1	--	--	--	--	--	--
Zfp809	uc009oof.2	chr9:22040980-22041097	118	Y						Y		N	28	--	--	14.8	14.6	-1.0	--	--	--	--	--	--
Zfp13	uc008ash.2	chr17:23721716-23721905	190	Y						Y		N	36	--	--	12.8	13.0	1.0	--	--	--	--	--	--
Ercc6	uc007sze.1	chr14:33355101-33355215	115	Y								Y	38	--	--	3.1	3.6	1.1	--	--	--	--	--	--
Tnfaip1	uc007kjr.2	chr11:78342265-78342360	96	Y			Y					N	16	--	--	6.5	8.6	1.3	--	--	--	--	--	--
Fam21	uc009djs.2	chr6:116191061-116191169	109	Y								Y	5	--	--	41.9	56.2	1.3	--	--	--	--	--	--
Sptbn4	uc009fwa.1	chr7:28180377-28180650	274	Y				Y	Y			N	42	--	--	2.6	3.6	1.4	--	--	--	--	--	--
Tnnt1	uc012ewu.1	chr7:4457602-4457736	135	Y								N	30	--	--	2.1	3.4	1.6	--	--	--	--	--	--
Flnb	uc007sek.1	chr14:8767880-8768447	568	Y							Y	Y	12	--	--	22.8	40.3	1.8	--	--	--	--	--	--
Ahnak	uc008goj.1	chr19:9064934-9065298	365	Y				Y	Y			N	7	--	--	24.0	125.7	5.2	--	--	--	--	--	--
Letm1	uc008xbi.1	chr5:34122223-34122253	31		Y	Y						Y	--	32	5	--	--	--	21.6	20.6	-1.0	18.0	5.1	-3.6
Mcoln1	uc009krq.1	chr8:3513301-3513555	255		Y	Y						Y	--	20	18	--	--	--	18.4	14.4	-1.3	10.6	4.8	-2.2
Mrps6	uc007zyu.1	chr16:92096292-92096397	106		Y	Y						Y	--	10	10	--	--	--	29.8	18.3	-1.6	24.8	13.8	-1.8

Mical2	uc009jgo.2	chr7:119398244-119398745	502		Y	Y				Y		N	--	18	100	--	--	--	71.7	51.2	-1.4	16.0	11.0	-1.5
Pkia	uc008oof.1	chr3:7368316-7368393	78		Y	Y		Y				N	--	7	32	--	--	--	57.5	82.1	1.4	231.3	171.3	-1.3
Drosha	uc007vib.2	chr15:12762977-12763144	168		Y	Y						Y	--	38	12	--	--	--	21.7	16.3	-1.3	10.3	8.2	-1.3
Plekha6	uc007cpu.2	chr1:135158452-135159587	1136		Y	Y						Y	--	30	10	--	--	--	6.4	7.5	1.2	0.4	0.4	-1.0
Ssbp2	uc007rjv.2	chr13:91678359-91678409	51		Y	Y						Y	--	15	11	--	--	--	9.5	10.5	1.1	1.9	1.8	-1.0
Ncs1	uc008jdq.1	chr2:31143004-31143039	36		Y	Y		3'UTR				N	--	9	85	--	--	--	93.9	71.9	-1.3	1.5	2.8	1.8
Zbtb16	uc009pim.1	chr9:48469634-48469707	74		Y	Y						Y	--	11	26	--	--	--	9.0	7.1	-1.3	4.9	13.8	2.8
Abr	uc007kft.1	chr11:76277793-76282079	4287		Y						Y	Y	--	12	--	--	--	--	59.7	20.4	-2.9	--	--	--
Abr	uc007kft.1	chr11:76281757-76282079	323		Y							Y	--	8	--	--	--	--	59.7	20.4	-2.9	--	--	--
Dlg3	uc009twm.2	chrX:97969004-97969071	68		Y							Y	--	11	--	--	--	--	17.0	6.2	-2.7	--	--	--
Ralgps2	uc007dcx.2	chr1:158746064-158746095	32		Y							Y	--	50	--	--	--	--	7.1	2.7	-2.6	--	--	--
Cacna1b	uc008ipe.1	chr2:24547480-24547585	106		Y							Y	--	83	--	--	--	--	7.6	3.0	-2.5	--	--	--
Pitpnm3	uc007jya.1	chr11:71870101-71870196	96		Y							Y	--	30	--	--	--	--	6.8	2.8	-2.4	--	--	--
Celf5	uc007gie.2	chr10:80932583-80932711	129		Y							Y	--	19	--	--	--	--	8.6	3.6	-2.4	--	--	--
Tctn2	uc008zqh.2	chr5:125053858-125057033	3176		Y						Y	Y	--	48	--	--	--	--	7.2	3.5	-2.1	--	--	--
Slit3	uc007ild.2	chr11:35131153-35132546	1394		Y							Y	--	50	--	--	--	--	10.3	5.2	-2.0	--	--	--
Slit3	uc007ild.2	chr11:35133328-35133517	190		Y							Y	--	20	--	--	--	--	10.3	5.2	-2.0	--	--	--
Slit3	uc007ild.2	chr11:35133856-35136003	2148		Y					Y		N	--	41	--	--	--	--	10.3	5.2	-2.0	--	--	--
Unc13a	uc009mei.2	chr8:74191228-74191271	44		Y							Y	--	38	--	--	--	--	30.1	17.8	-1.7	--	--	--
Kcnmb4	uc007hbw.1	chr10:115880828-115880968	141		Y							Y	--	18	--	--	--	--	43.6	30.0	-1.5	--	--	--
Add2	uc009cre.1	chr6:86041466-86041557	92		Y							Y	--	3	--	--	--	--	38.9	27.2	-1.4	--	--	--
Meis2	uc008lqt.2	chr2:115888892-115888923	32		Y						Y	Y	--	22	--	--	--	--	3.8	2.7	-1.4	--	--	--
Cdh22	uc008nxc.2	chr2:165008739-165008871	133		Y			Y				N	--	58	--	--	--	--	4.6	3.3	-1.4	--	--	--
Fxyd2	uc009pfv.1	chr9:45217129-45217256	128		Y							Y	--	69	--	--	--	--	3.6	2.7	-1.3	--	--	--
Rasal1	uc008zhr.1	chr5:121103275-121103516	242		Y							Y	--	24	--	--	--	--	12.9	9.6	-1.3	--	--	--
Rasal1	uc008zhr.1	chr5:121103779-121103888	110		Y							Y	--	14	--	--	--	--	12.9	9.6	-1.3	--	--	--
Lurap1l	uc008tjv.1	chr4:80599233-80599305	73		Y							N	--	10	--	--	--	--	22.0	16.5	-1.3	--	--	--
Aatk	uc007mrm.2	chr11:119869942-119870003	62		Y							N	--	9	--	--	--	--	13.4	10.3	-1.3	--	--	--
Arhgef4	uc007api.1	chr1:34860376-34860746	371		Y			Y				N	--	4	--	--	--	--	88.2	69.4	-1.3	--	--	--
Ap3b2	uc009iby.1	chr7:88635065-88635163	99		Y							Y	--	16	--	--	--	--	30.1	23.8	-1.3	--	--	--
St6galnac5	uc008rtu.1	chr3:152504152-152506379	2228		Y							Y	--	14	--	--	--	--	65.2	53.4	-1.2	--	--	--
Ppp3ca	uc008rmg.1	chr3:136339898-136340253	356		Y				Y			N	--	9	--	--	--	--	360.0	298.3	-1.2	--	--	--
Mrps27	uc007rpn.1	chr13:100128905-100132919	4015		Y						Y	Y	--	13	--	--	--	--	10.6	8.8	-1.2	--	--	--
Lrrc49	uc009pzf.2	chr9:60485456-60485476	21		Y		Y					N	--	19	--	--	--	--	10.9	9.2	-1.2	--	--	--
Kalrn	uc012aew.1	chr16:33995419-33995510	92		Y							Y	--	26	--	--	--	--	7.0	6.1	-1.1	--	--	--
R3hdm2	uc007hjl.2	chr10:126889703-126889747	45		Y		Y				Y	N	--	8	--	--	--	--	20.8	18.2	-1.1	--	--	--
Ntrk3	uc009hxh.2	chr7:85634947-85635385	439		Y							Y	--	17	--	--	--	--	15.7	13.8	-1.1	--	--	--
Src	uc008npb.1	chr2:157257346-157257451	106		Y			Y				N	--	60	--	--	--	--	22.1	19.5	-1.1	--	--	--
Cx3cl1	uc009mwx.1	chr8:97298977-97299173	197		Y							Y	--	7	--	--	--	--	137.2	121.7	-1.1	--	--	--
Ak5	uc008rtq.1	chr3:152298921-152306879	7959		Y					Y		N	--	5	--	--	--	--	92.0	82.1	-1.1	--	--	--
Tub	uc009jdf.1	chr7:116168888-116169121	234		Y						Y	Y	--	12	--	--	--	--	43.1	39.3	-1.1	--	--	--
Ip6k1	uc009roc.1	chr9:107940950-107941005	56		Y							Y	--	4	--	--	--	--	47.2	46.0	-1.0	--	--	--
Chga	uc007oui.1	chr12:103796508-103796675	168		Y						Y	Y	--	16	--	--	--	--	46.0	44.9	-1.0	--	--	--
Cd200	uc007zim.1	chr16:45390084-45390297	214		Y			3'UTR				N	--	8	--	--	--	--	33.7	33.1	-1.0	--	--	--
Slc45a1	uc008vxt.1	chr4:150004509-150004563	55		Y							N	--	46	--	--	--	--	11.5	11.7	1.0	--	--	--
Cluh	uc007kca.1	chr11:74463592-74463680	89		Y							Y	--	49	--	--	--	--	12.6	12.8	1.0	--	--	--
Sncb	uc007qpc.1	chr13:54867186-54867375	190		Y			Y				N	--	18	--	--	--	--	170.1	176.0	1.0	--	--	--
Sncb	uc007qpc.1	chr13:54867276-54867299	24		Y			Y				N	--	14	--	--	--	--	170.1	176.0	1.0	--	--	--
Sncb	uc007qpc.1	chr13:54867276-54867351	76		Y			Y				N	--	22	--	--	--	--	170.1	176.0	1.0	--	--	--
Rbfox1	uc007yce.2	chr16:5999507-5999571	65		Y			Y				N	--	8	--	--	--	--	10.2	10.7	1.0	--	--	--
Gabra1	uc007imf.1	chr11:41993978-41994160	183		Y			Y				N	--	16	--	--	--	--	72.3	78.8	1.1	--	--	--
Ssbp2	uc007rjv.2	chr13:91678217-91678409	193		Y							Y	--	5	--	--	--	--	9.5	10.5	1.1	--	--	--
Glis2	uc007xzw.1	chr16:4612002-4612527	526		Y					Y		N	--	54	--	--	--	--	2.3	2.6	1.1	--	--	--
Ank3	uc007fna.1	chr10:69338074-69340577	2504		Y						Y	Y	--	36	--	--	--	--	6.3	7.3	1.2	--	--	--
Rfx1	uc009mlv.2	chr8:86610298-86610343	46		Y							Y	--	41	--	--	--	--	3.2	3.8	1.2	--	--	--
Camk1g	uc007eel.1	chr1:195195061-195195146	86		Y			Y				N	--	71	--	--	--	--	9.5	11.7	1.2	--	--	--
Numbl	uc009fvq.1	chr7:28046027-28046126	100		Y							Y	--	31	--	--	--	--	35.3	43.6	1.2	--	--	--
Crlf1	uc009maj.1	chr8:73020450-73020595	146		Y							Y	--	73	--	--	--	--	5.6	7.0	1.3	--	--	--
Crlf1	uc009maj.1	chr8:73021096-73021195	100		Y							Y	--	73	--	--	--	--	5.6	7.0	1.3	--	--	--
AK083927	uc007vit.1	chr15:24914597-24914638	42		Y			ncRNA				N	--	100	--	--	--	--	0.8	1.0	1.3	--	--	--
Usp36	uc011yim.1	chr11:118147968-118148085	118		Y			Y				N	--	50	--	--	--	--	4.8	6.5	1.3	--	--	--
Pafah2	uc008vey.2	chr4:133957549-133957751	203		Y				Y			N	--	100	--	--	--	--	1.3	1.9	1.4	--	--	--
Rgs17	uc007egg.2	chr10:4430642-4430748	107		Y			Y				N	--	71	--	--	--	--	7.3	11.7	1.6	--	--	--
Rgs17	uc007egg.2	chr10:4432803-4432832	29		Y			Y				N	--	36	--	--	--	--	7.3	11.7	1.6	--	--	--
Uggt2	uc007uzj.2	chr14:119399951-119400172	222		Y							Y	--	33	--	--	--	--	2.1	4.2	1.9	--	--	--
Pard3	uc009nzf.2	chr8:130131568-130131715	148			Y						N	--	--	36	--	--	--	--	--	--	1.9	0.4	-5.1
Epn1	uc009ezx.1	chr7:5045736-5045833	98			Y						Y	--	--	27	--	--	--	--	--	--	44.6	9.8	-4.5
Sh3bgr	uc008acu.1	chr16:96425650-96425694	45			Y	Y					N	--	--	52	--	--	--	--	--	--	673.3	175.6	-3.8
Immp2l	uc007nll.1	chr12:42737559-42737641	83			Y						Y	--	--	100	--	--	--	--	--	--	10.3	3.8	-2.7
Abhd16a	uc008cfq.1	chr17:35228764-35231195	2432			Y					Y	Y	--	--	32	--	--	--	--	--	--	37.0	14.2	-2.6
Hps5	uc009gzg.2	chr7:54034808-54034837	30			Y	Y					N	--	--	62	--	--	--	--	--	--	1.3	0.5	-2.5
Ankrd9	uc007pcj.1	chr12:112217231-112219244	2014			Y		Y				N	--	--	25	--	--	--	--	--	--	12.2	5.0	-2.4
Ankrd9	uc007pcj.1	chr12:112223381-112223505	125			Y		Y				N	--	--	25	--	--	--	--	--	--	12.2	5.0	-2.4
Ankrd9	uc007pcj.1	chr12:112225136-112225342	207			Y			Y			N	--	--	25	--	--	--	--	--	--	12.2	5.0	-2.4
Ndufv2	uc008dgw.1	chr17:66447153-66447313	161			Y						Y	--	--	9	--	--	--	--	--	--	295.6	133.7	-2.2
Thoc7	uc007sge.1	chr14:14792641-14792730	90			Y		Y				N	--	--	27	--	--	--	--	--	--	104.7	47.5	-2.2
Rxrg	uc007dlb.2	chr1:169554495-169554572	78			Y		Y				N	--	--	58	--	--	--	--	--	--	31.1	14.5	-2.1
Afap1l1	uc008fcq.1	chr18:61903415-61906431	3017			Y					Y	Y	--	--	41	--	--	--	--	--	--	12.0	5.6	-2.1
Dysf	uc009cou.1	chr6:84105157-84129665	24509			Y					Y	Y	--	--	72	--	--	--	--	--	--	2.3	1.1	-2.1

Acyp2	uc007ihw.1	chr11:30525062-30525114	53		Y						N	--	--	5	--	--	--	--	--	--	430.3	210.4	-2.0
Ptpn3	uc008sya.2	chr4:57211358-57217778	6421		Y					Y	Y	--	--	47	--	--	--	--	--	--	11.2	5.9	-1.9
Sgcd	uc007ioz.1	chr11:46891238-46891319	82		Y						Y	--	--	44	--	--	--	--	--	--	19.5	10.4	-1.9
Ebpl	uc007ufv.2	chr14:61977135-61977212	78		Y						Y	--	--	56	--	--	--	--	--	--	2.9	1.6	-1.8
Vdac1	uc007ivm.1	chr11:52169253-52169334	82		Y		Y				N	--	--	5	--	--	--	--	--	--	966.9	538.7	-1.8
Kbtbd3	uc009obj.2	chr9:4312142-4312259	118		Y		Y				N	--	--	77	--	--	--	--	--	--	1.8	1.0	-1.8
Pxn	uc008zea.1	chr5:115965783-115966013	231		Y				Y		N	--	--	25	--	--	--	--	--	--	7.0	4.1	-1.7
Ubac2	uc007vao.1	chr14:122296381-122296454	74		Y						Y	--	--	32	--	--	--	--	--	--	16.9	10.3	-1.6
Ubac2	uc007vao.1	chr14:122302529-122302586	58		Y						Y	--	--	18	--	--	--	--	--	--	16.9	10.3	-1.6
Stau2	uc007ajn.2	chr1:16506829-16506878	50		Y		Y				N	--	--	71	--	--	--	--	--	--	7.6	4.7	-1.6
Stau2	uc007ajn.2	chr1:16506963-16507016	54		Y		Y				N	--	--	58	--	--	--	--	--	--	7.6	4.7	-1.6
Mlxipl	uc008zxt.1	chr5:135605679-135607059	1381		Y						Y	--	--	48	--	--	--	--	--	--	11.8	7.4	-1.6
Trappc9	uc007wbl.2	chr15:72691750-72693009	1260		Y				Y		N	--	--	16	--	--	--	--	--	--	4.6	2.9	-1.6
Scn4a	uc007lyu.1	chr11:106193962-106194079	118		Y						Y	--	--	48	--	--	--	--	--	--	56.0	35.8	-1.6
Scn4a	uc007lyu.1	chr11:106195208-106195444	237		Y			Y			N	--	--	48	--	--	--	--	--	--	56.0	35.8	-1.6
Cgrrf1	uc007thl.1	chr14:47471464-47471527	64		Y						Y	--	--	19	--	--	--	--	--	--	35.8	24.0	-1.5
Chd1	uc007skm.2	chr17:15859639-15860610	972		Y				Y		N	--	--	56	--	--	--	--	--	--	233.2	156.8	-1.5
Nedd4	uc009qqe.1	chr9:72534110-72534263	154		Y						Y	--	--	15	--	--	--	--	--	--	62.1	42.4	-1.5
Dnaja2	uc009mqa.1	chr8:88069042-88069176	135		Y	Y					N	--	--	8	--	--	--	--	--	--	90.9	62.8	-1.4
Abcc9	uc009epn.1	chr6:142609406-142611333	1928		Y				Y		N	--	--	36	--	--	--	--	--	--	23.1	16.2	-1.4
Irf2	uc009lqo.2	chr8:47825418-47825530	113		Y		Y				N	--	--	60	--	--	--	--	--	--	8.3	6.1	-1.4
Irf2	uc009lqo.2	chr8:47825622-47825753	132		Y				Y		N	--	--	60	--	--	--	--	--	--	8.3	6.1	-1.4
Trim55	uc008orw.1	chr3:19548304-19548357	54		Y						N	--	--	21	--	--	--	--	--	--	24.5	18.0	-1.4
Trim55	uc008orw.1	chr3:19549975-19550611	637		Y				Y		N	--	--	21	--	--	--	--	--	--	24.5	18.0	-1.4
Ddx23	uc007xne.1	chr15:98489783-98490025	243		Y		Y				N	--	--	16	--	--	--	--	--	--	26.5	19.8	-1.3
Rbm33	uc008wtz.1	chr5:28655922-28656000	71		Y						Y	--	--	24	--	--	--	--	--	--	4.5	3.4	-1.3
Coq10a	uc007hmk.1	chr10:127805754-127806329	576		Y		Y				N	--	--	27	--	--	--	--	--	--	110.9	83.3	-1.3
Coq10a	uc007hmk.1	chr10:127807663-127807871	209		Y			Y			N	--	--	27	--	--	--	--	--	--	110.9	83.3	-1.3
Nudt7	uc009nnw.2	chr8:116670105-116670153	49		Y						Y	--	--	32	--	--	--	--	--	--	19.4	14.8	-1.3
Agpat3	uc007fxp.1	chr10:77758613-77758725	113		Y		Y				N	--	--	47	--	--	--	--	--	--	5.9	4.6	-1.3
Tns1	uc011wmx.1	chr1:73962212-73962262	51		Y						Y	--	--	67	--	--	--	--	--	--	6.5	5.2	-1.3
Tns1	uc011wmx.1	chr1:73972343-73974169	1827		Y					Y	Y	--	--	18	--	--	--	--	--	--	6.5	5.2	-1.3
Clip4	uc008dna.2	chr17:72188337-72188416	80		Y						N	--	--	28	--	--	--	--	--	--	20.5	16.4	-1.3
Tmem117	uc007xjn.1	chr15:94460090-94460162	73		Y		Y				N	--	--	67	--	--	--	--	--	--	10.2	8.4	-1.2
Plcl1	uc007ban.2	chr1:55772638-55772665	28		Y						N	--	--	50	--	--	--	--	--	--	1.1	1.0	-1.1
Rsu1	uc008ijw.1	chr2:13089860-13089896	37		Y						N	--	--	26	--	--	--	--	--	--	17.5	16.6	-1.1
Ccndbp1	uc008lxg.1	chr2:120835744-120837169	1426		Y					Y	Y	--	--	12	--	--	--	--	--	--	14.2	14.0	-1.0
Fto	uc009mst.2	chr8:93840482-93840579	98		Y						Y	--	--	13	--	--	--	--	--	--	25.8	25.9	1.0
Erp44	uc008suy.1	chr4:48289614-48289658	45		Y						Y	--	--	16	--	--	--	--	--	--	17.1	17.1	1.0
Tctex1d2	uc007yyt.1	chr16:32420415-32420491	77		Y						Y	--	--	59	--	--	--	--	--	--	14.9	15.4	1.0
Eif4g3	uc008vkd.1	chr4:137619921-137620183	263		Y			Y			N	--	--	44	--	--	--	--	--	--	16.1	16.8	1.0
Sema4d	uc007qmo.1	chr13:51788502-51788562	61		Y		3'UTR				N	--	--	32	--	--	--	--	--	--	8.0	8.4	1.1
Psap	uc007fen.2	chr10:59750885-59750923	39		Y	Y					N	--	--	4	--	--	--	--	--	--	279.1	294.8	1.1
Pgpep1	uc009mbc.1	chr8:73182677-73182791	115		Y						Y	--	--	24	--	--	--	--	--	--	5.3	5.9	1.1
Sema3c	uc008wnl.1	chr5:17189822-17191102	1281		Y				Y		N	--	--	39	--	--	--	--	--	--	12.0	13.3	1.1
Npepps	uc007ldv.1	chr11:97131709-97131735	27		Y						Y	--	--	39	--	--	--	--	--	--	17.8	20.4	1.1
Tep1	uc007tlt.1	chr14:51444837-51446096	1260		Y					Y	Y	--	--	48	--	--	--	--	--	--	1.2	1.4	1.1
Zmynd8	uc008nyd.1	chr2:165706588-165706944	357		Y		Y				N	--	--	64	--	--	--	--	--	--	1.1	1.3	1.2
Lmcd1	uc009ddv.2	chr6:112242179-112242319	141		Y						Y	--	--	21	--	--	--	--	--	--	65.1	79.2	1.2
Txnrd1	uc007gjz.1	chr10:82325760-82325829	70		Y		Y				N	--	--	18	--	--	--	--	--	--	18.7	23.7	1.3
Mlip	uc009qtg.2	chr9:77154226-77154278	53		Y		Y				N	--	--	6	--	--	--	--	--	--	9.8	12.9	1.3
Mlip	uc009qtg.2	chr9:77155452-77155782	331		Y			Y			N	--	--	3	--	--	--	--	--	--	9.8	12.9	1.3
Tnrc6b	uc007wvt.1	chr15:80590061-80590225	165		Y					Y	Y	--	--	19	--	--	--	--	--	--	0.9	1.4	1.5
3110082I17Rik	uc009agk.1	chr5:139934472-139934722	251		Y		Y				N	--	--	17	--	--	--	--	--	--	5.4	8.6	1.6
Mad2l1bp	uc008cru.2	chr17:46286035-46286066	32		Y						N	--	--	100	--	--	--	--	--	--	11.3	18.3	1.6
AK158482	uc008esw.1	chr18:39546408-39546455	48		Y		Y				N	--	--	66	--	--	--	--	--	--	0.2	0.3	1.6
Slc16a1	uc008qui.1	chr3:104449950-104450202	253		Y		Y		Y		N	--	--	16	--	--	--	--	--	--	9.8	17.9	1.8
Sec63	uc007eyx.2	chr10:42546978-42546993	16		Y						N	--	--	65	--	--	--	--	--	--	5.2	10.9	2.1
Nxn	uc007kfp.2	chr11:76173830-76173975	146		Y			Y			N	--	--	25	--	--	--	--	--	--	0.6	1.3	2.3
Zbtb16	uc009pim.1	chr9:48469634-48473259	3626		Y					Y	Y	--	--	68	--	--	--	--	--	--	4.9	13.8	2.8
Chac1	uc008lts.2	chr2:119177373-119177910	538		Y					Y	Y	--	--	17	--	--	--	--	--	--	15.0	43.4	2.9
Lnpep	uc008apk.1	chr17:17713717-17713815	99		Y						Y	--	--	39	--	--	--	--	--	--	1.4	18.8	13.0
Gm5860	--	chr4:81729644-81729736	93		Y		ncRNA				N	--	--	67	--	--	--	--	--	--	--	--	--
Il17b	uc008fcl.1	chr18:61835751-61835856	106		Y			Y			N	--	--	100	--	--	--	--	--	--	0.0	5.2	>100
Rhox3h	uc012hgj.1	chrX:35077391-35077475	85		Y			Y			N	--	--	100	--	--	--	--	--	--	0.0	1.9	>100

Table S4. Tdp-43 cryptic exons found in mouse stem cell, muscle and neuron (refer to Excel Data Table). Inf.Ins., Inframe Insertion; Exon Ext., Exon Extension; NMD, Nonsense-mediated decay, PSI; Percent Spliced In.
